# Co-differentiation and enrichment of corneal endothelial cells and keratocytes from human pluripotent stem cells

**DOI:** 10.1038/s41598-025-03509-3

**Published:** 2025-05-28

**Authors:** Abhinav Reddy Kethiri, Pyry Grönroos, Ajai Suwaraj Chinnaiah Nagaraj, Heli Skottman

**Affiliations:** Eye Regeneration Group, Faculty of Medicine and Health Technology, Tampere University, Tampere, Finland

**Keywords:** Cornea, Stroma, Keratocytes, Endothelium, Pluripotent stem cell, Differentiation, Differentiation, Visual system

## Abstract

Corneal keratocytes (CK) and endothelial cells (CEnC) maintain corneal stromal transparency. Damage to these cell types can lead to visual impairment. Although corneal transplantation is effective, donor shortages limit its availability. Human pluripotent stem cells (hPSC) offer a promising alternative for generating corneal cell types. While hPSC-derived epithelial cells and CEnC are well-studied, attempts to differentiate CK from hPSC have received less attention. This study investigates the differentiation of hPSC-CK using defined culture conditions and approaches to enrich both CK and CEnC. hPSC were cultured on laminin 521 (LN-521) coatings and differentiated using transforming growth factor β (TGFβ) and glycogen synthase kinase-3 (GSK-3) inhibitors, along with retinoic acid (RA) with (mEn protocol) or without (En protocol) fibroblast growth factor-2 (FGF2). Differentiated cells were characterized using qPCR and immunofluorescence on days (D) 8, 10, and 13. CK enrichment utilized collagen-1-coated plates with keratocyte-specific media, while CEnC were purified through metabolic starvation. Results showed the formation of heterogenous cultures containing both CK and CEnC. CK-like cells expressed keratocan (*KERA*), lumican (*LUM*), paired box 6 (*PAX6*) and actin α2 (*ACTA2*) genes, with proteoglycan expression (lumican (LUM) and decorin (DCN)) increasing over time. En protocol however maintained a stable CK phenotype. Furthermore, enriched hPSC-CK were LUM^+^/DCN^+^/PAX6^−^/CD166^−^ and hPSC-CEnC were CD166^+^/ZO-1^+^. Using xeno-free, defined conditions, we differentiated both CK and CEnC from hPSC using a single protocol with further optimized enrichment methods for hPSC-CK and hPSC-CEnC purification, advancing cell differentiation techniques.

**Supplementary Information:**

The online version contains supplementary material available at 10.1038/s41598-025-03509-3.

## Introduction

The essential role of corneal transparency for clear vision is primarily attributed to the corneal stroma, which contains structurally organized collagen fibrils, interspersed corneal keratocytes (CK), and richly hydrated proteoglycans^1^. The corneal stroma is covered anteriorly by the epithelium, which acts as an environmental barrier, and posteriorly by the endothelium, which provides nourishment. While CK maintains the collagen matrix, the relative hydration of the stroma, which dictates the structural arrangement of the collagen, is preserved by the corneal endothelial cells (CEnC)^2^. Any disruption of corneal physiology can lead to visual impairment and blindness.

Corneal blindness poses a significant burden worldwide, often caused by infectious keratitis and ocular trauma^3^, leading to dysfunctional CK or CEnC. While corneal transplantation remains primary treatment option^3,4^, global corneal donor shortage limits the availability of the treatment^5^. To reduce the dependency on donor tissues, cell therapies such as the in vitro cultured human CEnC^6,7^ or corneal stromal stem cells^8,9^ (CSSC) have been used to treat corneal disorders. However, these rely on donor tissues. Therefore, alternative cell sources such as the human pluripotent stem cells (hPSC) including human induced pluripotent stem cells (hiPSC) could be used to produce different corneal cell types^10^.

A significant progress has been made to derive corneal epithelial and CEnCs from hPSC^11–13^. However, attempts to differentiate CK from hPSC have received less attention. Early efforts by Chan and co-authors demonstrated the potential to induce human embryonic stem cells (hESC) to differentiate towards neural crest cells (NCC) by co-culturing them with mouse stromal PA6 cell line. These NCC were subsequently cultured as pellets with significant expression of keratocan gene, typical marker for CK^14^. Similarly, Naylor and coauthors also used a two-step approach, differentiating NCC from hiPSC and further culturing them as either pellets or on human cadaver limbal rims, which promoted CK-specific gene expression and the CK phenotype^15^. Also, notable study by Joseph and co-authors reprogrammed the human donor corneal fibroblasts into iPSC and differentiated those to CK to model keratoconus disease condition^16^. Additional research has reported the differentiation of CK either from the hESC-NCC or from the iPSC by manipulating the substrate stiffness mechanobiology^17^. Despite these advancements, many approaches to differentiate CK from hPSC utilize xenogeneic feeder layers or Matrigel, complicating the transition to clinical applications. Thus, these challenges need to be solved for the successful use of hPSC-CK in regenerative medicine and corneal stromal therapies.

During the embryonic development of the human cornea, the surface ectoderm presumably becomes corneal epithelium. Subsequently, two waves of periocular mesenchymal (POM) cells, which consist of NCCs and mesoderm-derived progenitor cells^18^ invade the space between the corneal epithelium and lens vesicle. The first wave of POM contributes to the formation of the corneal endothelium, followed by the second wave that gives rise to the corneal stroma^19–21^. The factors responsible for driving this POM migration remains unclear. The surface ectoderm and POM cells exhibit complex, bidirectional signaling involving Wnt/β-catenin, TGFβ, retinoic acid (RA), and fibroblastic growth factor (FGF) pathways, which regulate the corneal epithelial cell fate^22^. Knockout studies of FGF receptor-2 (FGFR2) during mouse corneal development has revealed adverse effects not only on the corneal epithelium but also on stromal CK. Interestingly, FGFR2 signaling is crucial in the beginning of corneal epithelial development but not for the continuous survival of these cells^23^. Additionally, FGF2 (also known as basic(b) FGF) plays a significant role in corneal stromal wound healing by activating the CK proliferation^24^ and is essential for human CK survival^16^ and the secretion of proteoglycans^25^.

We have previously reported directed differentiation of CEnC from hPSC during which we have also observed the emergence of thick islands of mesenchymal-like cell clusters^26,27^. Although the method allows the production of hPSC-CEnC with typical CEnC characteristics, the differentiation efficacy should be increased due to these unidentified contaminating cell populations in the culture. The current study investigates the cell clusters in hPSC-CEnC cultures, the role of FGF2 in differentiating hPSC-CK and the enrichment of both hPSC-CEnC and hPSC-CK. Our findings provide increased understanding of the mesenchymal-like cell clusters in hPSC-CEnC differentiation cultures and potential solutions to enrich both the hPSC-CK-like cells and the hPSC-CEnC using the same differentiation protocol.

## Methods

### Differentiation of hPSC-CEnC and hPSC-CK-like cells

The previously established and characterized hPSC lines were used in this study including, hESC line Regea08/017 and hiPSC line WT001.TAU.bB2^26,28^ as well as AICS-0016-184 iPSC line form Allen Institute for Cell Science (Coriell Institute). All the experiments involving cell culture work were conducted on the site of Tampere University, Faculty of Medicine and Health Technology. The faculty has the approval of the National Authority Fimea (Dnro FIMEA/2020/003758) to conduct research on human embryos, and supportive statements from Regional Ethics Committee of the Expert Responsibility area of Tampere University Hospital have been obtained by the research group, granting the permissions to derive, culture, and differentiate hESC lines (R05116), and to establish and use hiPSC lines in ophthalmic research (R16116). No new cell lines were generated for this study.

The hiPSC were primarily utilized for all differentiation and enhancement experiments unless stated otherwise. As an initial step for differentiation with modifications, we utilized a previously established protocol for hPSC-CEnC with minor changes^26,27^. Differentiations were carried out on laminin (Cat# LN521-05, Human recombinant laminin 521, Biolamina) coated 6 or 12 well-plates (Cat# 3335 or 3336, Corning CellBIND, Corning) with plating density of 5000–30,000 undifferentiated cells cm^-^^2^.

**Fig. 1:**
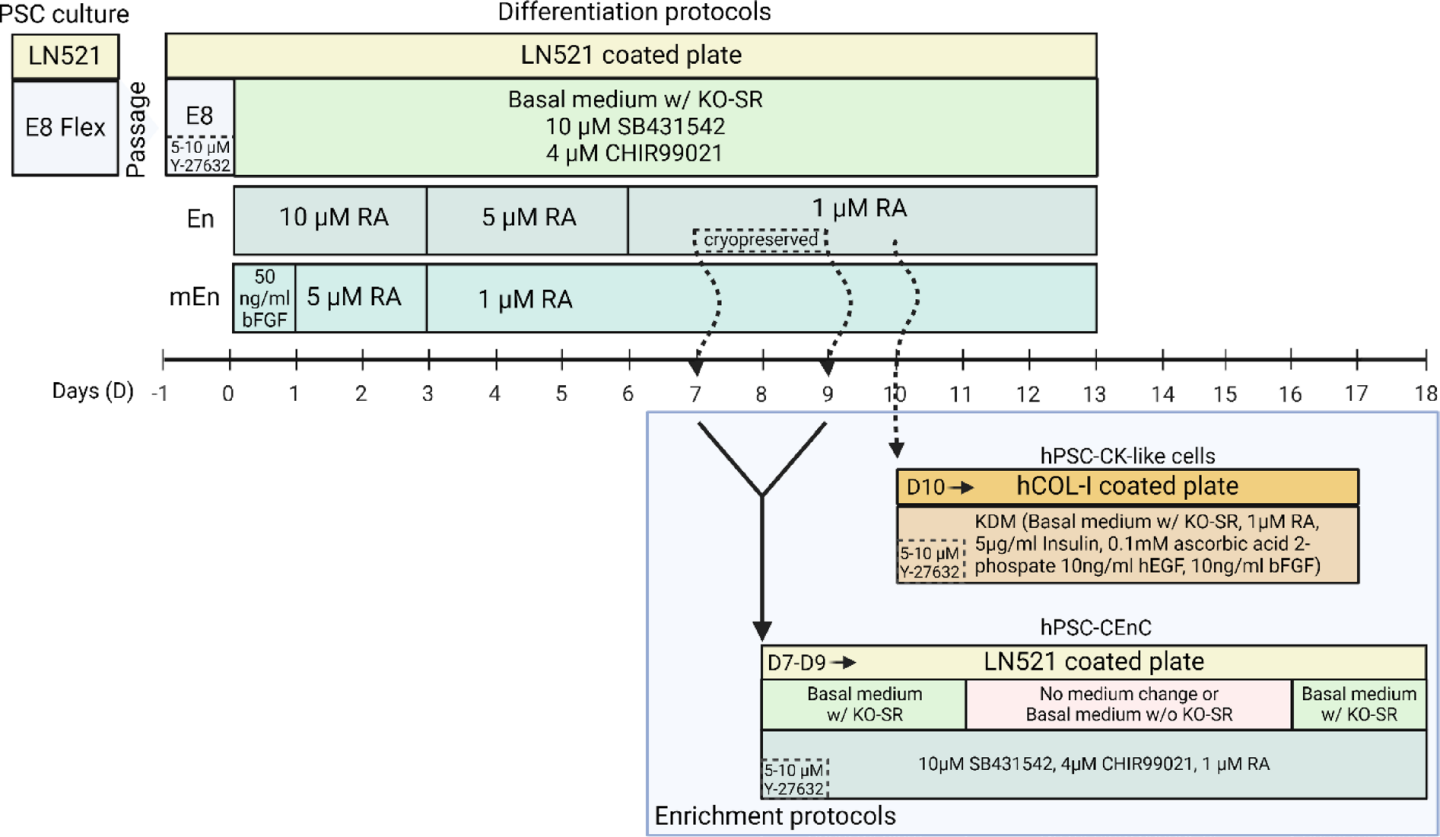
Schematic illustration of the differentiation and enrichment protocols used to derive hPSC-CEnC and hPSC-CK-like cells. “En” denotes the hPSC-CEnC protocol, while “mEn” refers to the modified hPSC-CEnC differentiation protocols. The enrichment protocols for hPSC-CEnC use cryopreserved cells collected on day(D) 7–9 from the “En” protocol. For hPSC-CK-like cells, the enrichment protocol uses freshly trypsinized cells collected on D10 from the “En” protocol. *PSC* pluripotent stem cell, *LN521* human recombinant laminin 521, *E8* essential 8 flex medium, *KO-SR* knockout serum replacement, *RA* retinoic acid, *hCOL-I* human type I collagen, *KDM* keratocyte differentiation medium, *hEGF* human epidermal growth factor, *bFGF* basic fibroblast growth factor. Created in https://BioRender.com.

hPSC were differentiated to CEnC and CK with both Endothelial (En) and the modified Endothelial (mEn) protocols (Fig. 1). Initially, hPSC were first seeded on the laminin coated plates and cultured with E8 medium (Essential 8 Flex Medium Kit, Cat# A2858501, Thermo Fisher Scientific) and 5–10 µM ROCK inhibitor (Y27632, Cat# 72304, STEMCELL Technologies) for 16–24 h. On days (D) 0 of differentiation, the medium was switched to serum free basal medium consisting of KnockOut Dulbecco’s Modified Eagle Medium (KO-DMEM) (Cat# 10829018, Gibco), 15% Knock-out serum replacement (KO-SR) (Cat# 10828028, Gibco), 2 mM Glutamine (GlutaMAX supplement, Cat# 35050038, Thermo Scientific), 0.1 mM 2-mercaptoethanol (Cat# 31350010, Gibco), 50 U ml^-1^ Penicillin-Streptomycin (Cat# 15140122, Gibco), 1% Non-essential Amino Acids (Cat# 11140035, Gibco) supplemented with 10 µM TGF-β inhibitor (SB431542, Cat# 72234, STEMCELL Technologies) and 4 µM GSK3 inhibitor/WNT pathway activator (CHIR99021, Cat# 72054, STEMCELL Technologies).

For En protocol previously described^26,27^, 10 µM of Retinoic acid (RA) (R2625, Sigma-Aldrich) was added to the above basal medium containing SB431542 and CHIR99021 on D0. During differentiation D3-6, 5 µM RA was added which was further decreased to 1 µM thereafter. For mEn protocol, En method was modified with an initial pulse of 50 ng/ml bFGF (Human FGF-basic, Recombinant Protein, PeproTech, Cat# 100-18B-50UG, Gibco) for the first 24 h and without RA to the basal medium containing SB431542 and CHIR99021. From D1-3, 5 µM RA was added followed by 1 µM RA until D13 of differentiation. Cells were cultured until D13 with cells obtained at different time points for analysis or enrichment. Phase contrast, light microscope (Nikon Eclipse TE2000-S with a DS-Fi1 camera, Nikon Corp.) was used to capture images of the cell morphology.

### Immunocytochemistry of the hPSC-CEnC and hPSC-CK-like cells

After differentiation, the phenotype of hPSC-CEnC and hPSC-CK used in the experiments were analyzed with immunofluorescence (IF). Protein markers identifying different cell types are listed in Supplementary Table 1. Briefly, cells were fixed with 4% paraformaldehyde (PFA, Cat# 15713-S, Sigma-Aldrich) for 15 minutes. Next, the cells were permeabilized for 10 minutes with 0.1% Triton X-100 (Cat# T8787, Sigma-Aldrich) followed by blocking with 3% bovine serum albumin (BSA) (Cat# A7906, Sigma-Aldrich) for 1 hour. Then the cells were first incubated with primary antibodies overnight at 4°C. The cells were washed and then treated with the respective secondary antibodies (Supplementary Table 2) for 1 hour at room temperature. The nuclei were counterstained with 1:1000 Hoechst 33342 (H3570, Invitrogen) with secondary antibody incubation or with 4’,6-diamidine-2’-phenylindole dihydrochloride (DAPI) in mounting medium (H-1000-10; Vector Laboratories). The images of stained cells were captured using a fluorescence microscope (Olympus IX51; Olympus) and processed using image editing software (Adobe Photoshop CC 2023; Adobe Systems).

### RNA extraction and PCR

Total RNA was extracted using RNeasy kit (RNeasy mini kit plus, Cat# 74136, Qiagen) protocol after trypsinizing the differentiated hiPSC (*n* = 3) at timepoints D8, D10 and, D13. Similarly, for enriched hPSC-CK and CEnC, RNA was extracted by Trizol (TRIzol™ Reagent, Cat# 15596026, Invitrogen) method and then was treated with DNase (DNase I, RNase-free, Cat# EN0525, Thermo Scientific) as per manufacturer’s protocol. RNA was quantified (NanoDrop 2000, Thermo Scientific) and converted into 200 µg of cDNA (High-capacity cDNA reverse transcription kit, Cat# 4374966, Applied Biosystems) that was either used for the reverse transcription (RT)-PCR (Supplementary Method 1) or RT-quantitative PCR (RT-qPCR).

For RT-qPCR, cDNA was analyzed using sequence-specific TaqMan Gene expression Assays (Thermo Scientific) for keratocan (*KERA*), lumican (*LUM*), decorin (*DCN*), Collagen, type I, alpha 1 (*COL1A1*), paired box 6 (*PAX6*), actin α2 (*ACTA2*), activated leukocyte cell adhesion molecule (*ALCAM*), ATPase Na+/K + transporting subunit alpha 1 (*ATP1A1*), cadherin 2 (*CDH2*), aquaporin 1 (*AQP1*), transcription factor AP-2 alpha (*TFAP2A*) and glyceraldehyde-3-phosphate dehydrogenase (*GAPDH*) (Supplementary Table 3). All the samples were run in triplicate reactions with the PCR system (QuantStudio 12 K Flex Real-Time PCR System, Applied Biosystems) and the results were obtained as cycle threshold values (C_T_) values by the software (QuantStudio 12 K Flex Software, Applied Biosystems). The quantitative expression of each gene-of-interest was calculated with the relative expression of the *gapdh* gene by applying the − 2^ΔΔCt^ method^29^. The expression was plotted in the graph with x-axis indicating the time points or genes and the y-axis showing the relative fold change.

### Enrichment of hPSC-CK-like cells

For hPSC-CK enrichment, 12-well plates (CellBIND) were coated as per manufacturer instructions with 75 µg/ml of human collagen-1 (hCOL1) (OptiCol Human Collagen Type I, Cat# M16S, Cell Guidance Systems) diluted in 1x PBS containing Mg^2+^ and Ca^2+^ (Cat# 14040174, Gibco). Cells differentiated with the En protocol were trypsinized at D10 and then all the cells were replated on the hCOL1 plates to ensure no CK-like cells were lost from the heterogenous cultures. A modified keratocyte differentiation medium^30^ (KDM) containing basal medium (similar to hPSC-CEnC or CK differentiation above), 1 µM RA, 5 µg/ml Insulin (Insulin solution human, Cat# I9278, Sigma-Aldrich), 0.1 mM L-Ascorbic acid 2-phosphate (Cat# A8960, Sigma-Aldrich), 10 ng/ml hEGF (Human EGF, Animal-Free Recombinant Protein, PeproTech, Cat# AF-100-15, Gibco) and 10 ng/ml bFGF was used to culture hPSC-CK-like cells. Initially, 5 µM ROCK inhibitor was added to KDM until D1 after which it was removed and cultured further for 6 more days (Fig. 1).

### Cryopreservation and thawing of hPSC-CEnC

For cryopreservation, cells were harvested at day 7–9 of the En protocol differentiation using TrypLE Select (Cat# 12563029, Thermo Fisher) dissociation enzyme and incubated for 3–6 min at + 37 °C. Subsequently, the hPSC-CEnC were detached gently by trituration, filtered through 40 μm cell strainer (Falcon Cell Strainer Sterile, Cat# 352340, Corning) and centrifuged for 5 min at 300 g. The cells were resuspended in cryomedium consisting of basal medium with 40% KO-SR and 10% dimethyl sulfoxide (DMSO, Cat# D2650, Sigma-Aldrich) in 2 ml cryogenic storage vial (Cat# 72.380, Sarstedt). After cryopreservation, the cells were thawed and passaged on LN521 coated 6 or 12 well-plates at 200 000–300 000 cells cm^-^^2^ in basal medium supplemented with 10 µM SB431542, 4 µM CHIR99021, 1 µM RA and 10 µM ROCK inhibitor. After 24 h, ROCK inhibitor was removed from the medium.

### Enrichment of hPSC-CEnC with metabolic starvation

Thawed hPSC-CEnC were cultured for 3 days until the cell culture gained full confluency and with polygonal CEnC. The cells were then either left without medium changes or the medium was changed to KO-SR-free medium (serum starvation) for 4–6 days. After starvation period, the dead cells were detached with gentle trituration and the alive hPSC-CEnC were left for differentiation medium with 1 µM RA up to 2 days (Fig. 1).

### Statistical analysis

Statistical analysis was performed with GraphPad Prism software (v9.0.0 GraphPad Software Inc.). Normal and lognormal distribution of the data was determined by the Kolmogorov-Smirnov and Shapiro-Wilk tests. For qPCR analysis, either the Friedman or Kruskal-Wallis test with Dunn’s multiple comparison correction method or the Mann-Whitney U test was applied. A p-value of ≤ 0.05 was considered statistically significant. Each ‘n’ represents a biological replicate, with at least three technical replicates per measurement.

## Results

### Identification of hPSC-CK-like cells during hPSC-CEnC differentiation

**Fig. 2:**
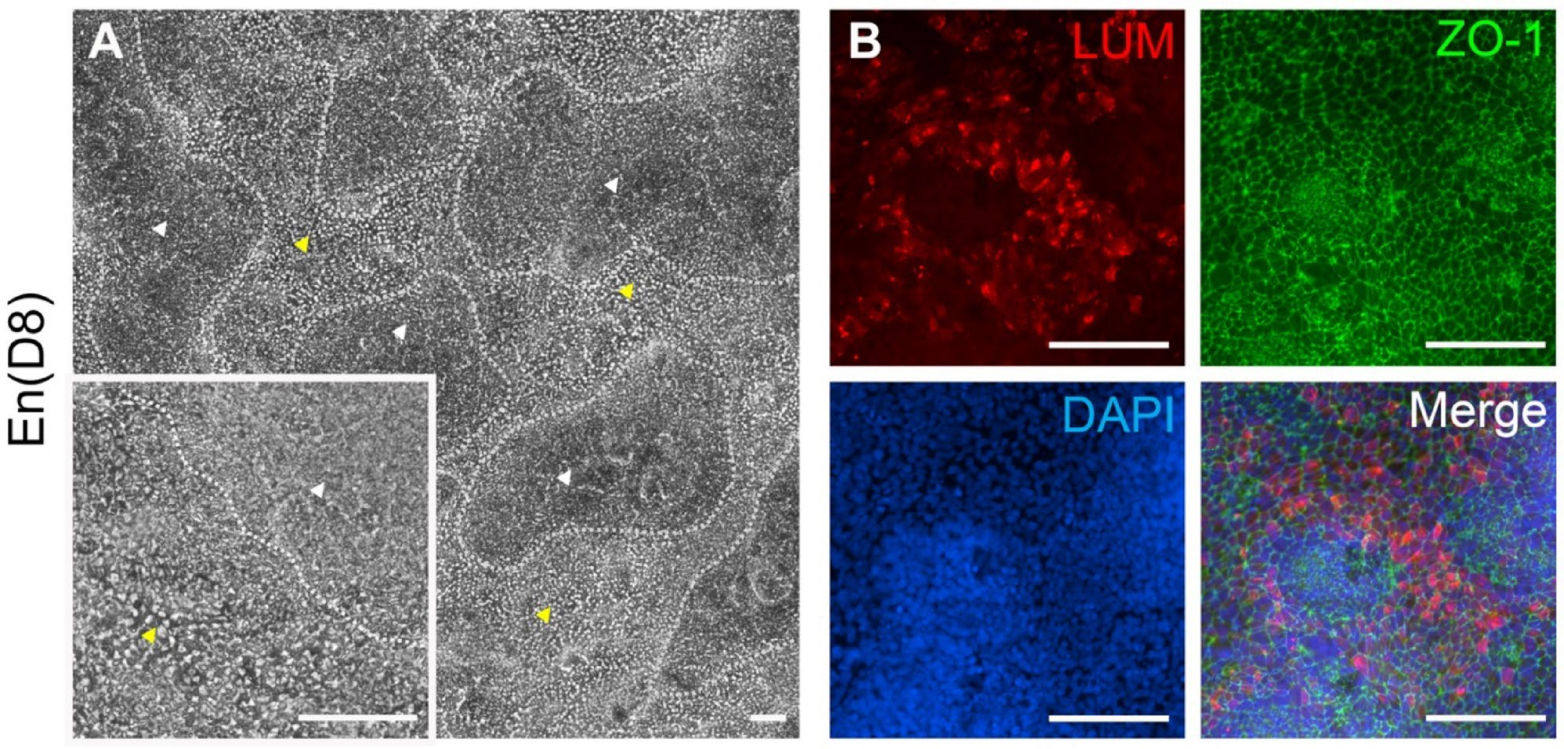
Cell clusters in differentiation cultures of hPSC-CEnC. (**A**) With the En protocol several polygonal hPSC-CEnC (areas with yellow arrowheads) are present at day(D) 8 along with the overgrowth of the mesenchymal-like cell clusters (areas of the grey dotted lines with white arrowheads). (**B**) Immunofluorescence of hPSC-CEnC at D8 shows the presence of lumican (LUM) expressing cells (red color) in the regions of cell clusters and co-expression of ZO-1 (green color). DAPI represents nuclei (blue color) and “Merge” represents all the colors merged. Data shown is representative images of the hiPSC (WT001.TAU.bB) differentiation to CK-like cells (*n* = 4) with two technical replicates. Objective magnification: (**A**) 4x and 10x. (**B**) 20x. Scale bar: 100 μm.

By differentiating hPSC using small molecules (SB431542, CHIR99021) and RA, we previously established the differentiation method for hPSC-CEnC expressing CEnC markers, i.e., CD166, Na^+^/K^+^-ATPase and ZO-1^26,27^. However, we have also observed a side-population of mesenchymal-like cells that consistently emerged within in the hPSC-CEnC cultures, competing with the CEnC growth and forming cell clusters by D8 (Fig. 2A). Interestingly, with additional IF staining we verified lumican (LUM) expression, a key proteoglycan marker for CK, in these clustering cells which exhibit characteristics similar to CK-like cells (Fig. 2B). This finding motivated us to characterize these cells further and also to increase the number of CK-like cells.

### Low seeding densities of hPSC were ineffective in differentiating into CK-like cells

**Fig. 3:**
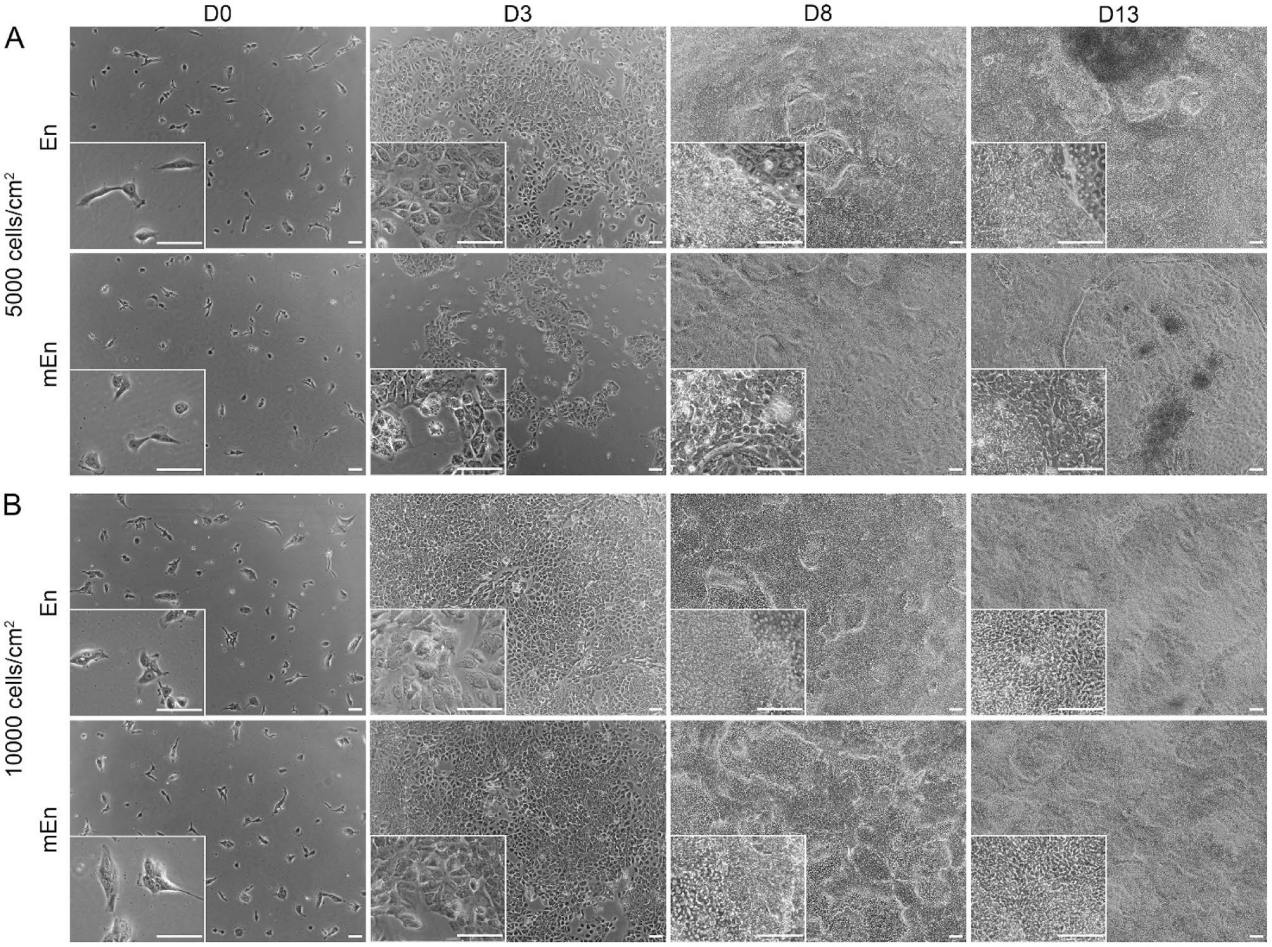
Differentiation of hPSC in low cell seeding densities. Phase contrast image shows the differentiated hiPSC with En and mEn protocol. (**A**) Cell density of 5000 cells cm^-^^2^ were not confluent enough at day(D) 3 of the differentiation. By D8-13, cell clusters were sparsely visible with more polygonal cells. (**B**) Cells seeded at 10,000 cells cm^-^^2^ however were confluent by D3 and developed more cell clusters at D8-13. Data shown here is representation of hiPSC (WT001.TAU.bB) differentiation to CK-like cells (*n* = 3). Objective magnification: 4x and 10x. Scale bar: 100 μm.

**Fig. 4:**
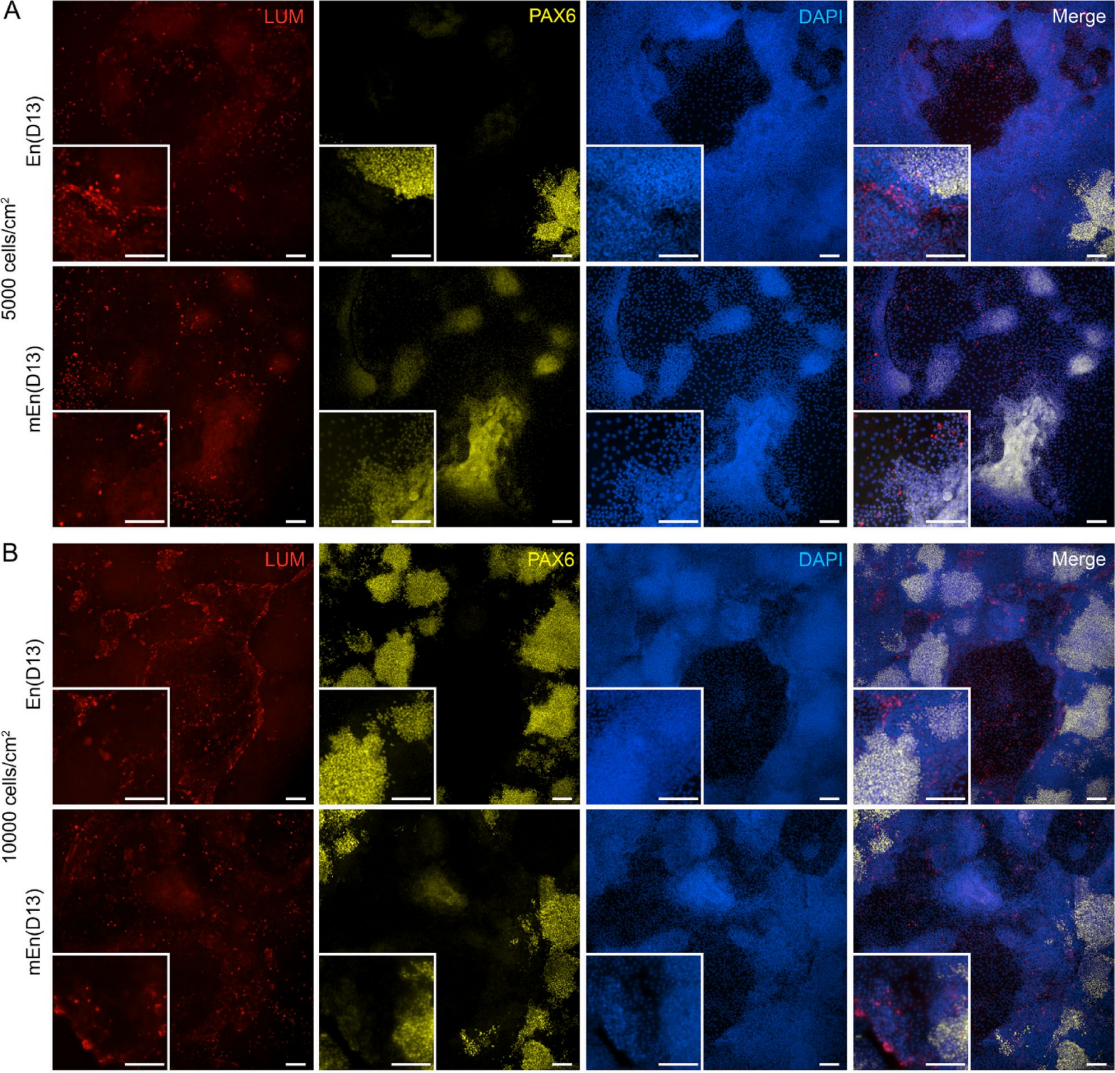
Characterization of hPSC-CK-like cells at low cell seeding densities. (**A**) Immunofluorescence indicate the minimal expression of lumican (LUM) and PAX6 expressing cells in hPSC-CK at day(D) 13 for 5000 cells cm^-^^2^ (5 K) cell seeding density. (**B**) Even though cell seeding density of 10,000 cells cm^-^^2^ (10 K) had developed cell clusters (as seen in DAPI; blue), the lumican (LUM; red) expressing cells were not sufficient in number for the enrichment. On the contrary, PAX6 (yellow) seems to have higher in expression in 10 K as evident from the cell clusters, compared to 5 K. Data represent findings from hiPSC (WT001.TAU.bB) differentiation to CK-like cells (*n* = 3). Objective magnification: 10x and 20x. Scale bar: 100 μm.

Next, we set up different experimentations to increase the differentiation of CK-like cells. First, to determine whether the formation of the CK-like cell clusters is dependent on the high cell confluency and overcrowding, we examined the effects of lower cell seeding densities of hPSC differentiation to the clusters of CK-like cells. In these experiments, the hPSC were cultured at different seeding densities of 5000 cells cm^− 2^ (5 K) and 10,000 cells cm^− 2^ (10 K). These cell densities were tested both with the En as well as modified mEn protocols (Fig. 1). At D3, it was observed that the cultures gained sufficient confluency at the cell density of 10 K, while those with the 5 K cell density did not. Interestingly by D13, both of the used cell densities developed cell clusters, but the cell culture with density of 5 K exhibited sparse clusters with prominent polygonal cells in both En and mEn protocols (Fig. 3A and B). Additionally, IF analysis revealed that the lower density 5 K cultures contained less PAX6 and also LUM expressing cells as compared to 10 K cultures. Although the 10 K cultures displayed a higher overall number of cell clusters, the number of LUM-expressing cells was not sufficient for further enrichment (Fig. 4A and B).

### Effect of modified protocol to stimulate the differentiation of CK-like cells

**Fig. 5:**
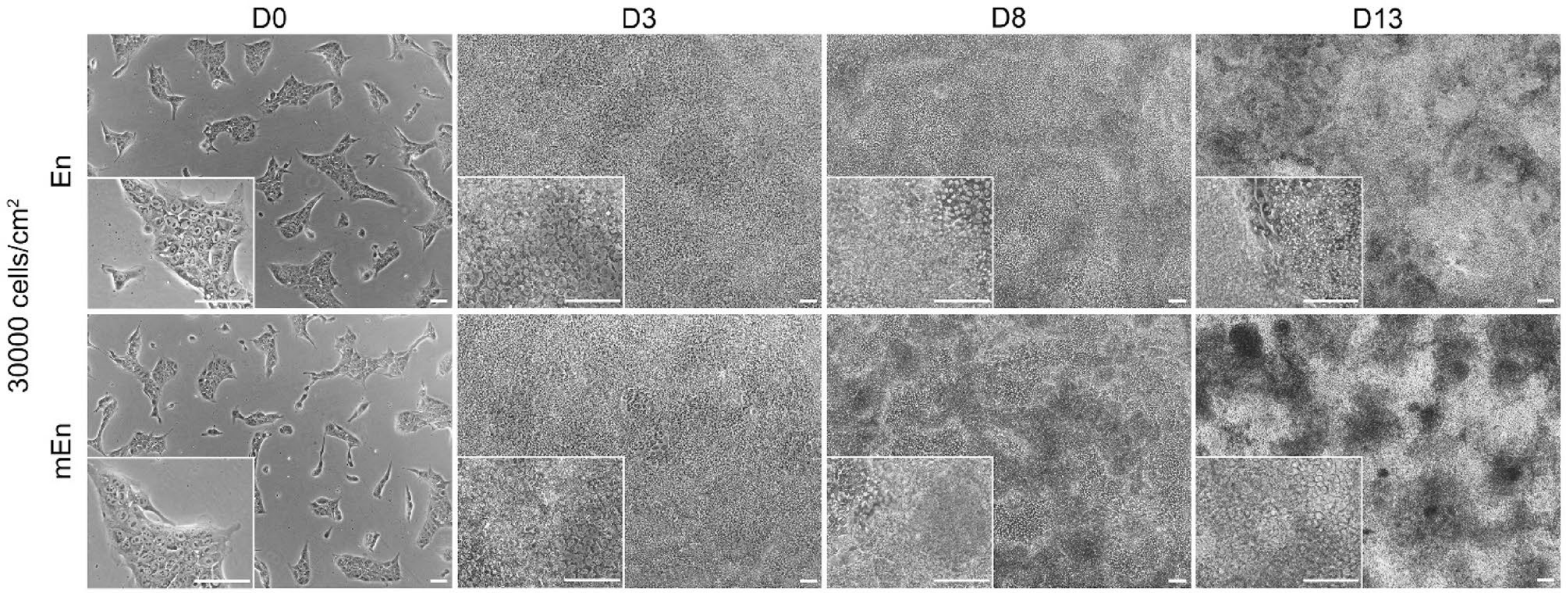
Differentiation of hPSC into CK-like cells with high seeding densities. Phase contrast image panel shows the hPSC seeded at a density of 30,000 cells cm^-^^2^. Cells were confluent by day 3 (D3) with both En and mEn protocols. Cell clusters began to develop by D8 and increase by D13. Individual cell morphologies were however not clear in the cell clusters and only a few areas showed polygonal CEnC. Data shown are the representative images of the hiPSC (WT001.TAU.bB) differentiated to CK-like cells (*n* = 4). Objective magnification: 4x and 10x. Scale bar: 100 μm.

Since the low cell seeding densities could not effectively enhance the differentiation of CK-like cells, we decided to culture hPSC at 30,000 cells cm^− 2^ (30 K) cell densities to facilitate further differentiation and characterization using both En and mEn protocols. Next our focus was on the different RA concentrations between En and mEn protocol as well as bFGF supplementation in the mEn protocol. By using both of these protocols, at D3 of differentiation, cells were already confluent and continued to develop cell clusters by D8 during which cells attached to the plate exhibited polygonal CEnC morphology and the clusters had mesenchymal-like characteristics. Further by D13, the clusters had increased in size and developed multiple cell layers (Fig. 5 and Supplementary Fig. 1).

**Fig. 6:**
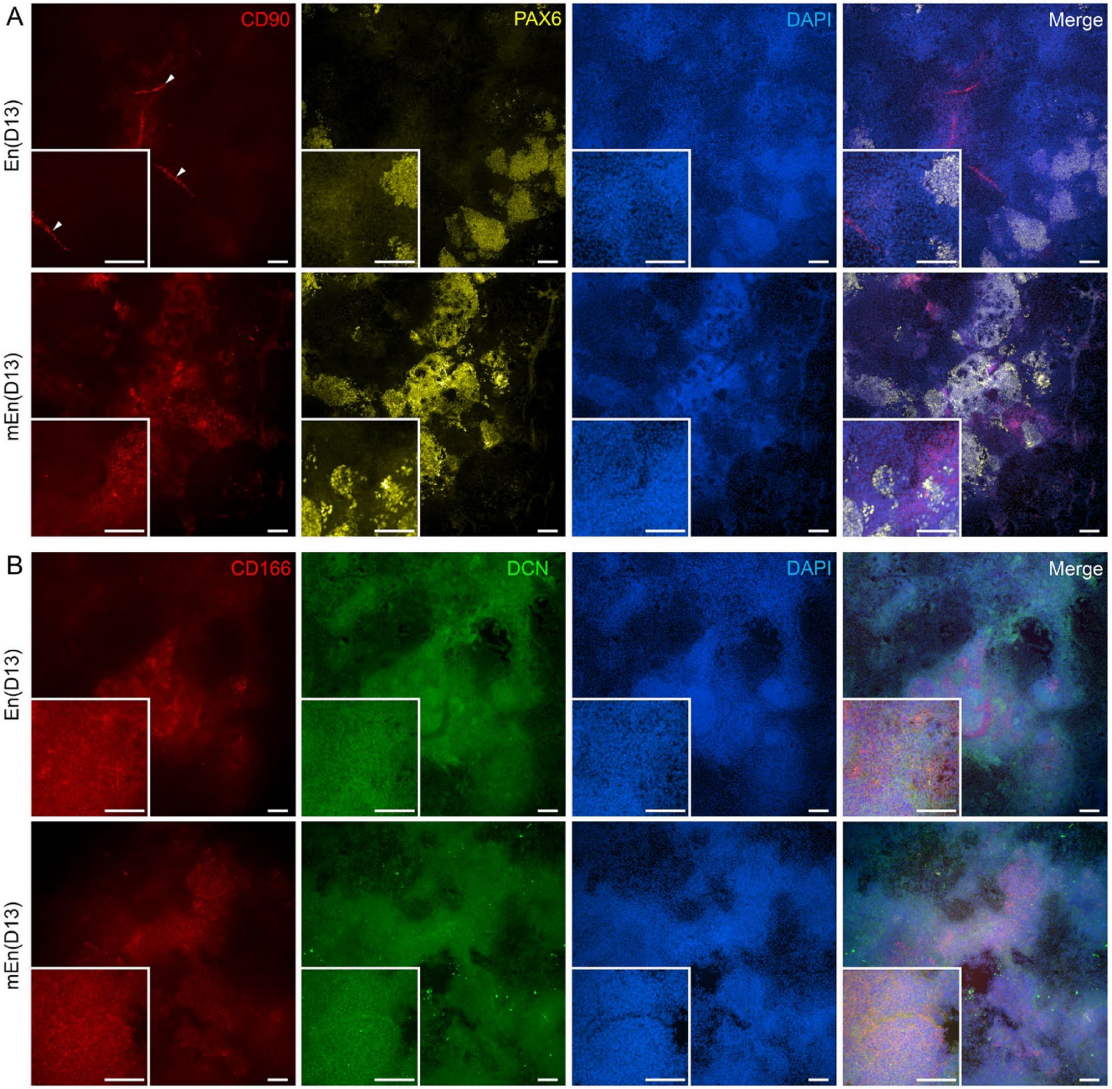

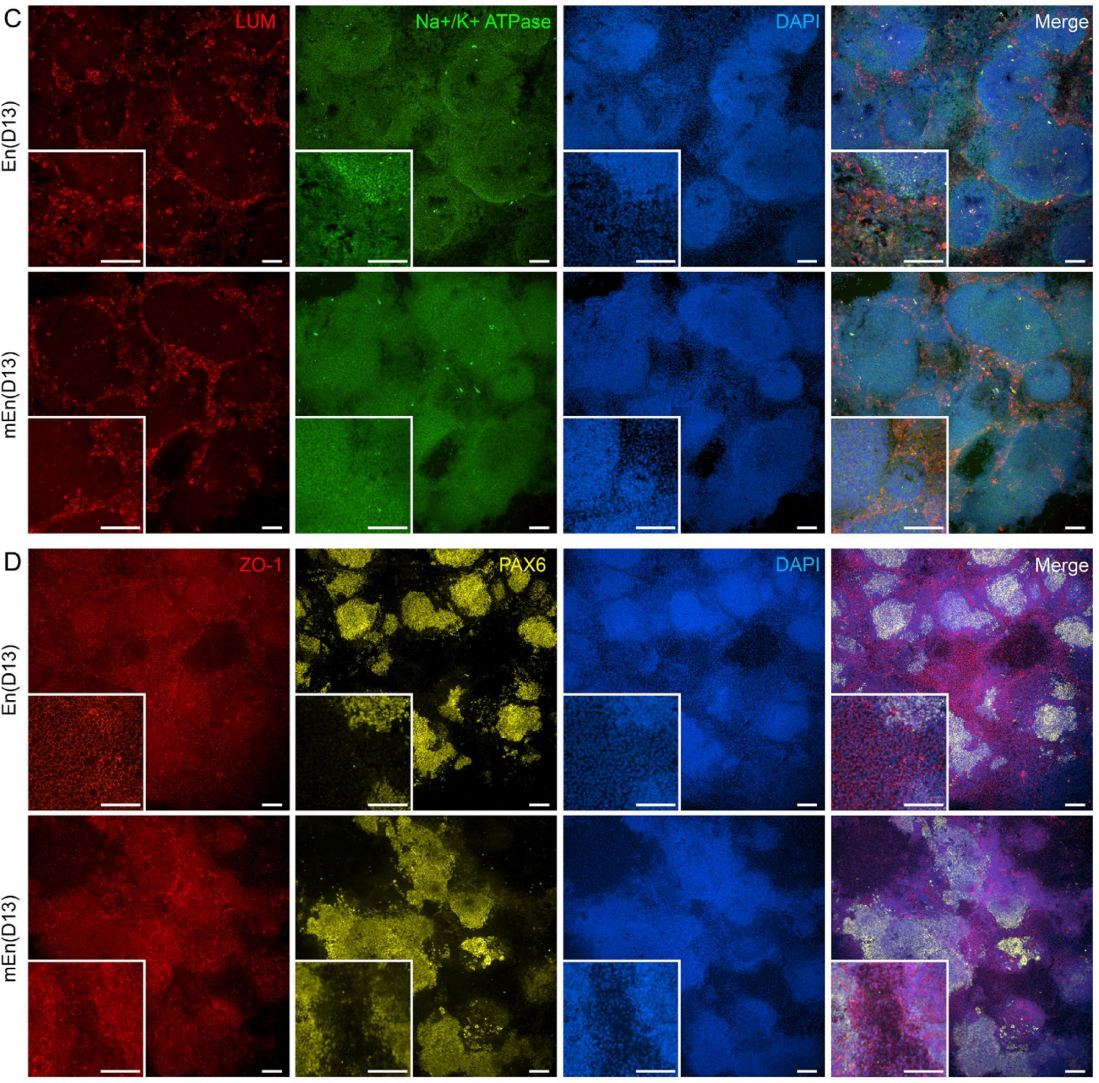
Expression of CK and CEnC specific markers in hPSC differentiated CK-like cells. IF analyses show the expression of CD90 (red) and PAX6 (yellow) (**A**), CD166 (red) and decorin (DCN; green) (**B**), lumican (LUM; red) and Na^+^/K^+^-ATPase (green) (**C**) and ZO-1 (red) and PAX6 (yellow) (**D**) in both En and mEn protocols after 13 days (D13) of differentiation. Abundant expression of the proteoglycans (LUM and DCN) was observed. Additionally, CD90 was expressed in cells with fibroblastic morphology and ZO-1 was expressed in cells of polygonal morphology, suggesting CEnC. White arrow heads indicate cells with fibroblast-like morphology. Data shown is representative images of the hiPSC (WT001.TAU.bB) differentiated to CK-like cells (*n* = 4) with two technical replicates in each. Objective magnification: 10x and 20x. Scale bar: 100 μm.

Next the hPSC-CK characteristics was assessed by IF that revealed the expression of key stromal cell markers, CD90 and eye field marker PAX6, in both En and mEn cultures at D13 (Fig. 6A). The cells also demonstrated expression of proteoglycans (LUM and decorin (DCN)) along with CEnC markers CD166, Na^+^/K^+^-ATPase and ZO-1 (Fig. 6B, C and D), indicating heterogenous cell types. A similar expression pattern of these markers was also observed at intermediate timepoints of D8 (Supplementary Fig. 3A, B, C and D) and D10 (Supplementary Fig. 3E, F, G and H), as well as in hESC differentiated to CK (Supplementary Fig. 4). Of note, undifferentiated hPSC did not express LUM but exhibited high CD90 expression, indicating that CD90 cannot be considered a CK-specific marker in hPSC differentiation cultures (Supplementary Fig. 2A and B).

**Fig. 7:**
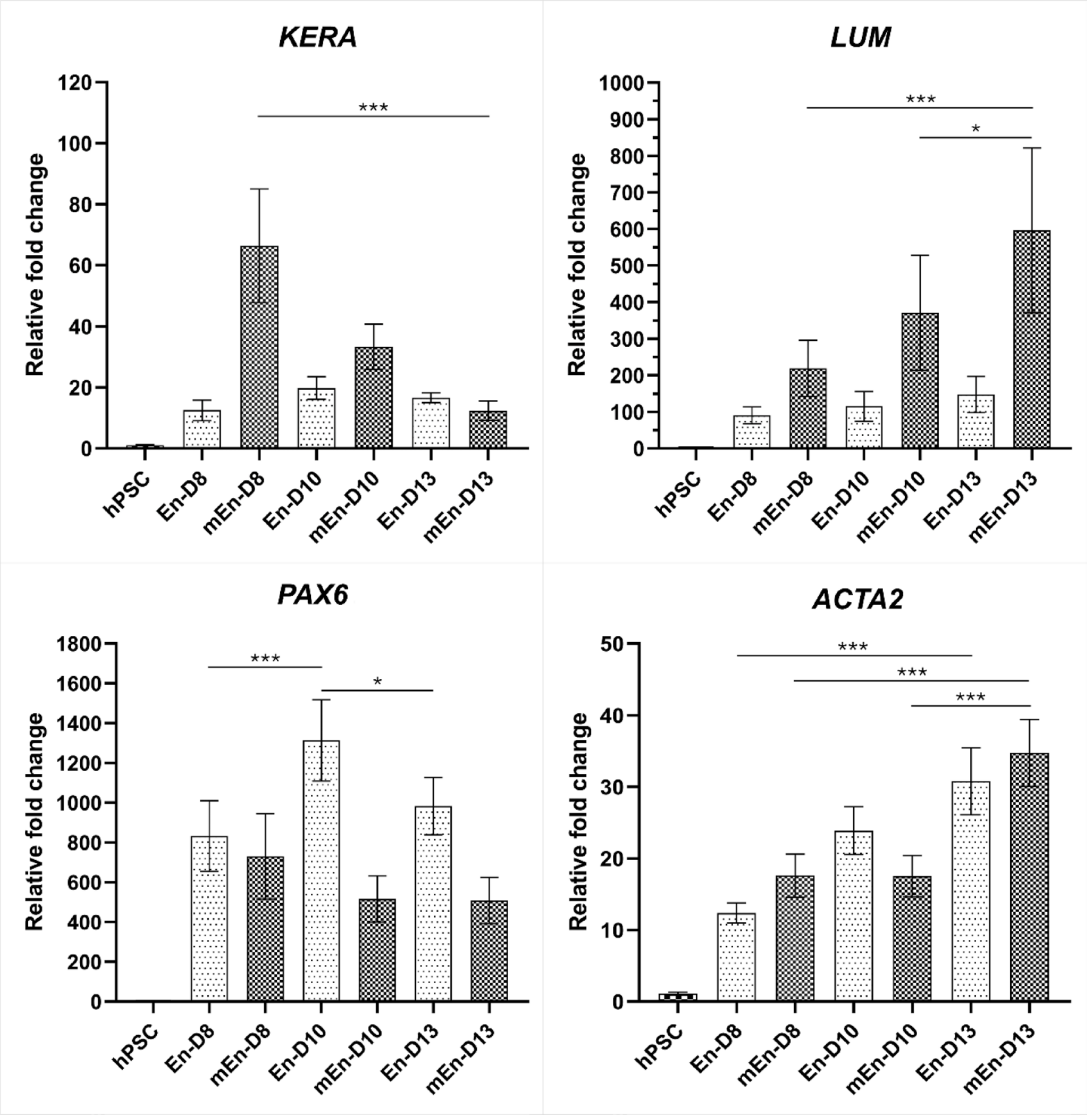
Gene expression analyses of the hPSC differentiated to CK-like cells. Gene expression analysis at days (D) 8, 10, and 13 for both En and mEn protocols indicated the increased expression of keratocan (*KERA*), lumican (*LUM*), *PAX6*, and *ACTA2* when compared to the undifferentiated hPSC. Friedman test to compare multiple means with Dunn’s method of pairwise comparison shows the relative fold changes of the genes at D8, 10, and 13 were significant (*p ≤ 0.05; *** *p* ≤ 0.001). Data represented as the mean relative fold changes of the gene with standard error in hiPSC (WT001.TAU.bB) differentiated to CK-like cells (*n* = 3).

As the initial IF analysis did not reveal any difference between the two tested differentiation protocols (En and mEn), the gene expression analysis was further performed to detect possible differences at transcriptional level. The initial analysis with RT-PCR revealed the similar gene expression pattern of the corneal stromal related genes in both En and mEn protocol (Supplementary Fig. 5). Further, RT-qPCR analyses were included to quantify the gene expression. The overall expression level of analyzed genes (*KERA*,* LUM*,* PAX6* and *ACTA2*) were significantly increased (*p* ≤ 0.001) throughout the differentiation with both En and mEn method when compared to the undifferentiated hPSC. However, there was no clear difference in gene expression levels between the two tested protocols. Based on the analyses in the En protocol, the relative fold change of *KERA* (En-D8: 12.48 ± 3.40; En-D13: 16.66 ± 1.61) and *LUM* (En-D8: 90.31 ± 23.22; En-D13: 147.6 ± 49.55) remained similar from D8 through D13. However, in the mEn protocol, the expression of *KERA* decreased (mEn-D8: 66.38 ± 18.57; mEn-D13: 12.39 ± 3.18; *p* ≤ 0.001) while *LUM* increased (mEn-D8: 218.5 ± 76.64; mEn-D13: 596.2 ± 225.5; *p* ≤ 0.001) from D8 through D13. Additionally, *PAX6* expression was significantly high at D13 in both En (En-D13: 983.1 ± 144.2; En-D10 vs. D13: *p* ≤ 0.05) and mEn (mEn-D13: 508.8 ± 116.4) methods. Further, smooth muscle actin (*ACTA2*) had increasing expression trend in both En (En-D8: 12.39 ± 1.38; En-D13: 30.79 ± 4.67; *p* ≤ 0.001) and mEn (mEn-D8: 17.61 ± 3.01; mEn-D13: 34.74 ± 4.66; *p* ≤ 0.001) methods (Fig. 7).

### Heterogeneity and co-expression of cell specific markers

**Fig. 8:**
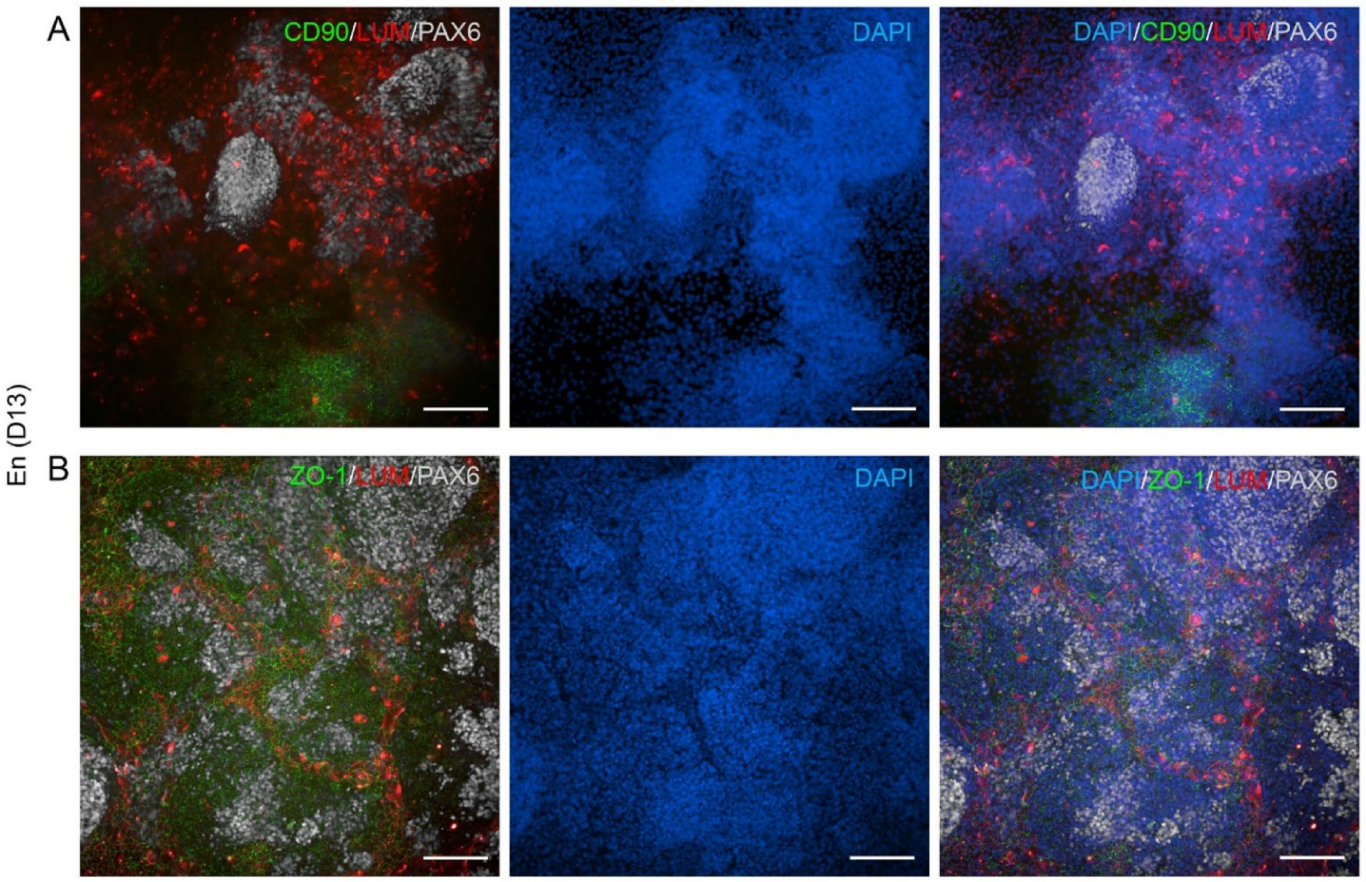
Heterogeneity of hPSC-CK-like cell cultures: (**A**) Cell clusters exhibit distinct expression patterns of PAX6 (white), CD90 (green), and lumican (LUM; red) in the hPSC-CK-like cell cultures on day (D) 13 of the En protocol. (**B**) ZO-1 (green) expression is uniform across the culture and tends to co-express with both LUM and PAX6. Data shown is representative images of the hiPSC (WT001.TAU.bB) differentiated to CK-like cells (*n* = 3). Objective magnification: 20x. Scale bar: 100 μm.

With the presence of both CEnC (CD166) and CK (proteoglycans) markers in hPSC differentiation cultures, we investigated the correlation of the protein expression with the cell morphology, distribution, and co-expression of these markers in different cell types. It was observed that PAX6-positive cells were predominantly located within cell clusters and were minimal in the monolayer. LUM-expressing cells were found surrounding these PAX6 clusters, while CD90-expressing cells were localized in different regions of the culture (Fig. 8A). These markers did not co-express within a single cell type but were distinct groups of cells with separate expression profiles. Additionally, a sub-population of CD90-expressing cells had distinct fibroblastic cell morphology in hPSC-CK cultures (Fig. 6A, Supplementary Fig. 3A, 3E, 4E and 4I) indicating mesenchymal cell characteristics. ZO-1, however, showed widespread expression throughout the culture, including within the cell clusters where it co-expressed with both LUM and PAX6 markers (Fig. 8B) indicating diversified cell types in the differentiated cultures.

### Enrichment of CK-like cells with collagen-1 substrate

**Fig. 9:**
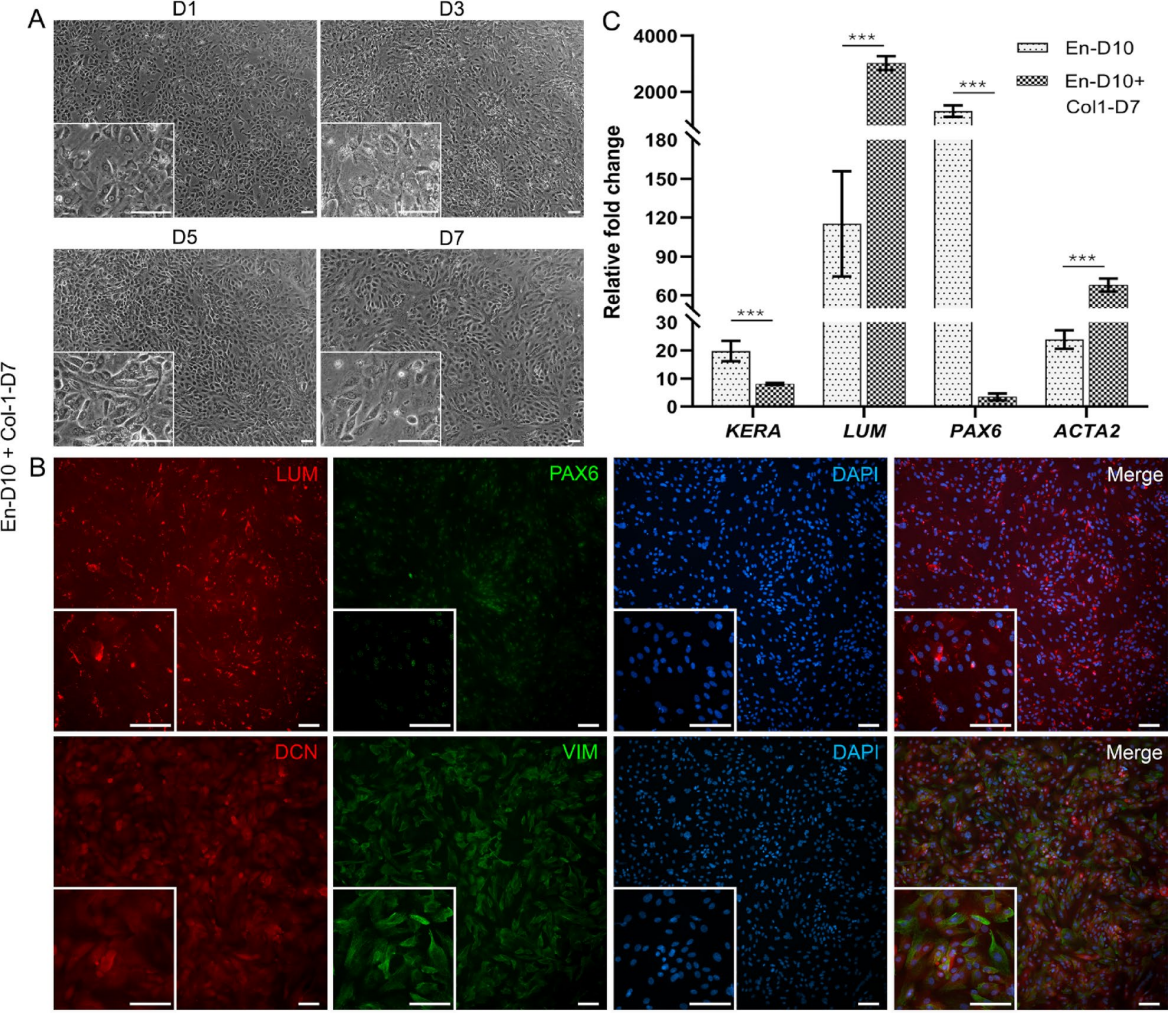
hPSC-CK-like cell enrichment on collagen-1 coated plates. (**A**) hPSC-CK-like cells on collagen-1 coated plates cultured for 7 days (D7) in keratocyte differentiation medium (KDM). Fibroblastic morphology of the cells began to appear at D3 that had become prominent by D7. (B) IF shows the abundant expression of key proteoglycans (lumican (LUM; red) and decorin (DCN; red)) along with vimentin (VIM; green) and low PAX6 (green) expression. Objective magnification: (**A**) 4x and 10x. (**B**) 10x and 20x. Scale bar: 100 μm. (**C**) The relative fold changes of *LUM* and *ACTA2* were significantly higher, while *KERA* and *PAX6* decreased in enriched hPSC-CK-like cells compared to hPSC-derived CK cells differentiated using the En protocol on Day 10 (En-D10). Mann-Whitney U test was performed to compare the ranks between the groups of a gene. Significant relative fold changes were indicated as ***p ≤ 0.001. Data shown is representative images of the hiPSC (WT001.TAU.bB) differentiated to CK-like cells (*n* = 3).

As the IF analyses clearly indicated the multiple cell types in hPSC-CK-like cell cultures, it was imperative to segregate the required CK. Therefore, we explored if cell substratum and media changes would enrich CK-like cells. To assess the impact of using different culture media, we switched the medium of the hPSC-CK cultures (En protocol) to KDM on D10 and continued culturing until D17. Phase contrast images at D17 reveal a fibroblast-like morphology in cultures containing KDM, contrasting with those maintained without KDM (Supplementary Fig. 6A). IF analysis further showed increased LUM and decreased CD90 expression in En-D10 and KDM-D7 cultures compared to En-D17, highlighting KDM’s influence on the differentiation and phenotypic characteristics of hPSC-CK-like cells (Supplementary Fig. 6B). However, KDM alone did not sufficiently reduce the heterogeneity within hPSC-CK cultures. Therefore, we combined KDM with a hCOL1 substrate coating to further enrich CK-like cells. Phase contrast images of cells indicate the cells were confluent already on D1 because of the high seeding density. Further by D5, cells had increased fibroblast-like morphology which became prominent by D7 (Fig. 9A). Additionally, IF of D7 cells on hCOL1 reveals notable increased expression of LUM, DCN and vimentin (VIM) with low PAX6 expression (Fig. 9B). Furthermore, a few cells expressed CD90, Na^+^/K^+^-ATPase, and α-smooth muscle actin (αSMA), with no CD166 expression and sparse ZO-1 expression (Supplementary Fig. 7). This expression pattern suggests the absence of hPSC-CEnC and presence of more purified, enriched hPSC-CK-like cells.

To validate the robustness of the IF findings, we conducted gene expression analysis on enriched hPSC-CK-like cells and compared their expression profiles with hPSC-derived CK cells differentiated using the En protocol on Day 10 (En-D10). The expression of *LUM* significantly increased after hPSC-CK enrichment (En-D10: 115.18 ± 40.55 vs. En-D10 + Col-1-D7: 3020.85 ± 250) while *PAX6* (En-D10: 1313.88 ± 204.33 vs. En-D10 + Col-1-D7: 3.45 ± 1.26) and *KERA* (En-D10: 19.76 ± 3.66 vs. En-D10 + Col-1-D7: 8.04 ± 0.35) showed decreased expression. Further, *ACTA2* expression significantly increased in enriched hPSC-CK cells (En-D10: 23.88 ± 3.34 vs. En-D10 + Col-1-D7: 67.95 ± 5.09) (Fig. 9C). Overall, enriched hPSC-CK cells exhibited significantly higher expression of proteoglycans and reduced expression of CEnC-related genes compared to hPSC cells (Supplementary Fig. 8) indicating successful CK-like cell enrichment.

### Enrichment of hPSC-CEnC with starvation culture

**Fig. 10:**
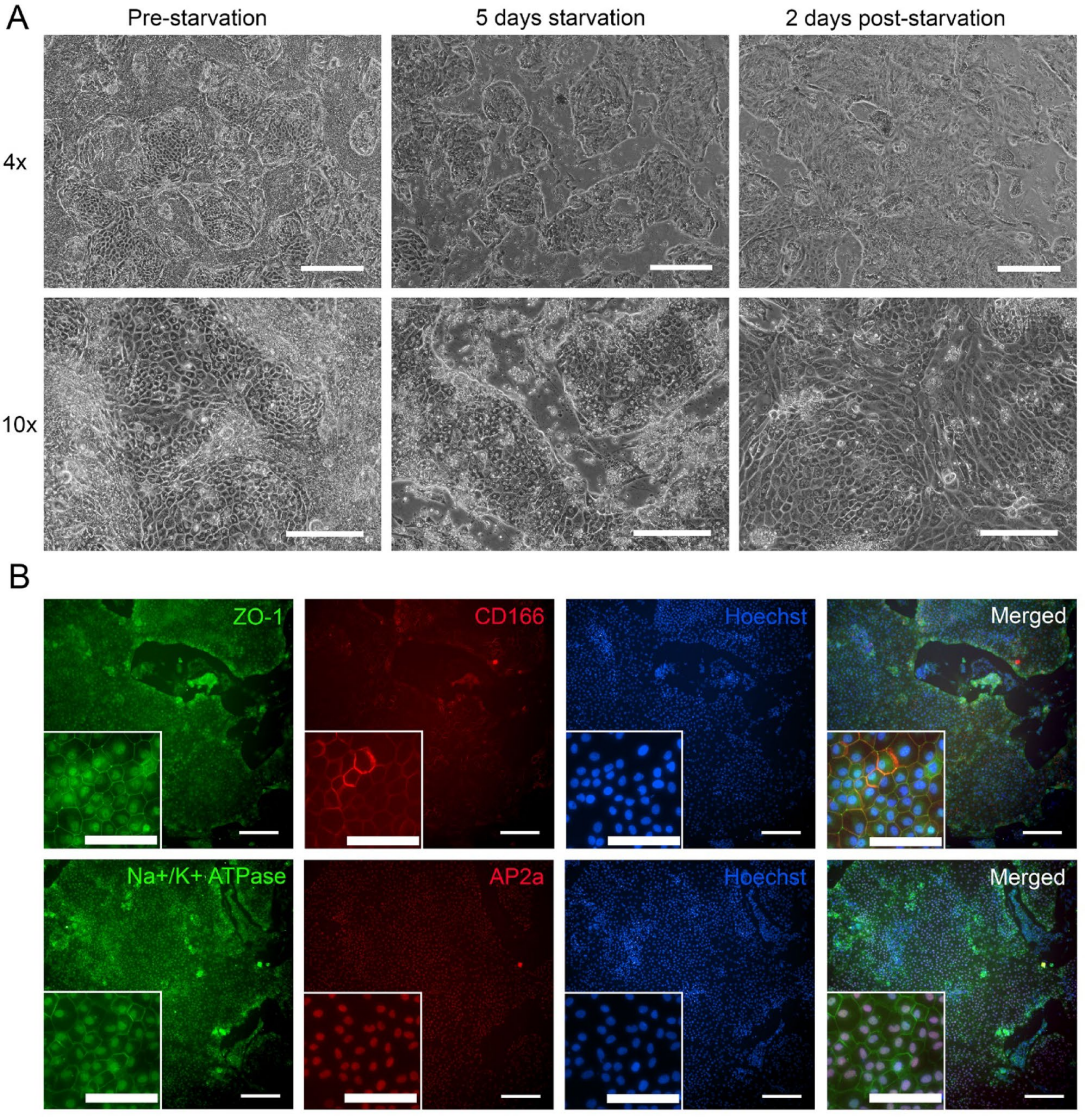
hPSC-CEnC enrichment by starvation method. (**A**) Phase contrast images of the cell culture before the start of the starvation period, after the starvation period of 5 days and 2 days post-starvation in normal En medium. (**B**) Immunofluorescence images of the cells 2 days after the starvation period in normal medium. ZO-1 (green) is localized in tight junctions, CD166 (red) in lateral membrane as well as Na^+^/K^+^-ATPase green). The majority of the survived cells expressed AP2α (red) in the nucleus. Data shown is representative images of the hESC differentiated to CEnC-like cells using medium starvation without medium changes. Number of biological replicates *n* = 3. Objective magnification: (**A**) 4x and 10x. (**B**) 10x and 20x. Scale bars: 200 μm and in magnified images, 100 μm.

As the heterogenic culture clearly indicated that the clusters of mesenchymal-like cells were more proliferative than the CEnC, we decided to test if the low nutrient environment would enhance CEnC by cleaning the culture from the more actively proliferating cell clusters. This was conducted via starvation for which cells were left without medium changes for 4–6 days or the medium without KO-SR was used for 4–6 days. These two methods showed no major differences between each other (Supplementary Fig. 9). In these starvation conditions, the hPSC-CEnC clearly tolerated the starvation environment much longer compared to other cell populations in the culture. The hPSC-CEnC remained as adherent monolayer with polygonal morphology while the majority of other mesenchymal-like populations detached (Fig. 10A). Based on the IF analyses, the hPSC-CEnC retained their typical protein marker expression (CD166, Na^+^/K^+^-ATPase, ZO-1 and AP2α) after the starvation period (Fig. 10B). Additionally, the localization of the N-cadherin marker indicates that the hPSC-CEnCs preserved their structural integrity (Supplementary Fig. 10). The gene expression analysis revealed a slight increase in CEnC-related markers CD166, ATP1A1, and CDH2, with a significant increase in AQP1, particularly under starvation conditions without KO-SR. The CK marker LUM also showed a slight increase. Additionally, the NCC marker TFAP2A, which is shown to be positive for hPSC-CEnCs differentiated with the current protocol^26^, had increased expression after starvation, indicating a relatively larger population of hPSC-CEnCs in the culture post-starvation (Supplementary Fig. 11). However, when the culture conditions were shifted back to the En medium, the remaining mesenchymal-like cells started to proliferate again in couple of days as evident in the time lapse imaging (Supplementary Fig. 12).

## Discussion

In this study, our aim was to develop a direct and defined method to differentiate CK cells from hPSC. Based on the knowledge of the cornea development^19–21^, our study hypothesized that hPSC could be differentiated into CK using our previously established xeno-free and defined hPSC-CEnC differentiation method (En) as a starting point^26,27^. Towards this goal, we first identified the mesenchymal-like cell clusters among hPSC-CEnC cultures and interestingly, based on the IF analyses, the cell clusters clearly indicated the presence of LUM positive CK-like cells (Fig. 2). This is similar to the report by McCabe and co-authors, who have described the formation of cell clumps in hESC derived CEnC that may be the early neural crest progenitors^31^. We have also previously demonstrated the expression of POM markers, FOXC1 and PITX2 in these cell clusters during the hPSC-CEnC differentiation^26^. Additionally, our differentiated hPSC cultures demonstrated the CEnC differentiation first by D6-8 and followed by increased amount of cell clusters with LUM-expressing cells during D8-13. This is in line with the embryological development of the human cornea with endothelium developed first and stroma later^20^.

Next, our aim was to test if different modifications for the En protocol including lower cell density as well as RA concentration modifications together with FGF2 supplementation (mEn) could affect the differentiation rate of the hPSC-CK-like cells. In both protocols, extending the differentiation time beyond D7-8 consistently led to the emergence of cell clusters (Figs. 2A and 5) similar to our previous reports^26^ suggesting a significant degree of culture heterogeneity. Additionally, it was observed that the lower seeding densities (5 K and 10 K) were also able to form cell clusters (Fig. 3) suggesting that clustering was not dependent on over confluency but rather on the differentiation induction under both En and mEn conditions. However, the lower seeding density clusters did not yield an adequate number of LUM-expressing cells and thus higher cell densities were used for further experiments (Fig. 4). As also reported with other hPSC differentiation methods towards different cell lineages^32,33^ we found that the high seeding density of 30 K was optimal, producing CK-like cells with robust LUM and DCN expression, as identified by IF (Fig. 6B and C). This higher cell density may provide a better microenvironment for hPSC-CK differentiation as reported with neuronal differentiation^34^. Interestingly also, a high expression of PAX6, a key transcriptional factor for neural crest migration during corneal development^35,36^, was detected in hPSC-CK cultures, primarily within cell clusters surrounded by LUM-expressing cells (Figs. 6 and 9), indicating the potential presence of migrating NCCs. Initially we considered that the CD90 positive cells in cultures could represent CSSC, an important stem cell population identified in the adult human cornea^37,38^ and may give rise to CK. However, both undifferentiated hiPSC and hESC also expressed high levels of this well-established stem cell marker (Supplementary Fig. 2A and 2B), thus specificity for CSSC cannot be confirmed. On a positive note, the CD90-positive cells on D8, 10, and 13 exhibited a distinct fibroblastic morphology (Fig. 6A and Supplementary Fig. 3A, 3E, 4E and 4I), clearly different from the morphology of undifferentiated hPSC and from that of PAX6- or LUM-expressing cells (Fig. 8A). Thus, further studies are needed to confirm if transient CSSC-like cells could be identified during the hPSC-CK differentiation process.

To further detect any difference in the En and mEn methods, gene expression analyses were conducted. These results indicated the stable *KERA* and *LUM* expression in cells produced with the En protocol, whereas cells generated with mEn protocol had decreased *KERA* and increased *LUM* expression, highlighting the potential differences in cell phenotype stability between the two protocols (Fig. 7). It has been previously reported with the LUM knockout studies in mice that LUM regulates the KERA expression proportionally with restoration of *KERA* expression by overexpressing *LUM*^39^. Our mEn protocol however shows inverse relationship between the expression level of *LUM* and *KERA* that warranted further investigation. This may suggest that while both protocols can induce CK-like characteristics, En protocol may support a more stable CK phenotype. Moreover, the consistently high levels of *PAX6* in both protocols through D13 highlight the robustness of ocular lineage commitment^40^. Further, the increased expression of *ACTA2* gene through D13 in both the protocols also indicated the possibility of endothelial-mesenchymal transitions^41^ evident by the increased cell clusters after D6-8 of hPSC differentiation. Altogether, our results indicated that both En and mEn protocols successfully differentiated hPSCs into CK-like cells without clear difference between the protocols. Thus, we chose to continue with the En protocol, as it offers the potential to generate both CEnC and CK-like cells using a single method.

Due to the heterogenous nature and mixed population of hPSC-CEnCs and hPSC-CK-like cells, we decided to further investigate if it would be possible to enrich both cell populations. Prior research has shown that primary CK characteristics can be enhanced in substrate-free cultures^42^, and therefore, previous attempts to differentiate CK from hPSC have utilized culturing cells as pellets to promote CK phenotype^14,15,43^. Studies have also demonstrated that collagen-rich extra cellular matrix influences the behavior and characteristics of CK^44,45^. Furthermore, KDM also plays an important role in maintaining CSSC or hPSC-CK^14,15,43^, while COL1-rich substrate promoted CK phenotype retention and inhibited myofibroblast transition of CK^46^. In our study, when hPSC-CK cultures were switched to KDM at En-D10, IF showed increased LUM expression but with the presence of other cell types. Additionally, IF analysis on D17 (Supplementary Fig. 6B) revealed decreased LUM-positive cells with En protocol, suggesting that an optimal time window between D8-10 of hPSC differentiation would be beneficial for enhancement of CK. Therefore, we combined hCOL1 coating with KDM for the hPSC-CK enrichment.

Cells differentiated with the En protocol until D10, followed by seeding on hCOL1 with KDM displayed high LUM and DCN expression (Fig. 9B), with a complete absence of CD166-positive hPSC-CEnC (Supplementary Fig. 7). Furthermore, the reduced expression of PAX6 in hCOL1 cultures suggests a shift toward the CK lineage, as primary CK are reported to have minimal or no expression of PAX6^47^. Similarly, gene expression analyses of enriched hPSC-CK-like cells revealed significantly higher *LUM* expression when compared to En-D10 hPSC-CK. The expected decrease in *PAX6* expression further supports CK lineage commitment. However, the decreased *KERA* expression may be attributed to increased *ACTA2* levels in enriched hPSC-CK (Fig. 9C), resembling the expression pattern observed in serum-induced differentiation of primary CK^48^. Additionally, the upregulation of proteoglycans and reduced expression of CEnC-related genes in enriched hPSC-CK confirms successful CK-like cell enrichment. These findings provided insight into the important role of hCOL1 contribution to the promising hPSC-CK enrichment strategy.

Lastly in this study, our aim was to test if further purification of the hPSC-CEnC would be feasible with culture conditions modifications. Based on literature, serum starvation has been routine procedure to prepare cells for different experimental settings including but not limited to cellular stress response studies^49^, autophagy and apoptosis studies^50–52^ and it has been shown that serum-nutrient starvation induces cell death^53^. In previous studies, primary hCEnC have been also cultured two days in serum-free medium without major drawbacks in cell viability^54^. Also, in our previous study^26^ the medium of cultured primary hCEnC were changed only twice a week indicating the good tolerance of low nutrient environments. It is well known that starvation reduces basal cellular activity and makes the population of proliferating cells more homogenous, since they withdraw from the cell cycle to enter the quiescent G0/G1 phase^55,56^. Since the mature hCEnC are arrested in G1 phase^57^, we hypothesized and investigated if our hPSC-CEnC also have better ability to withstand starvation-induced stress compared to the rapidly proliferating mesenchymal-like cell populations that often contaminate CEnC differentiation cultures. The starvation period effectively purified the hPSC-CEnC population from mesenchymal-like cells (Fig. 10), with the hPSC-CEnCs maintaining their structural integrity in both starvation methods, as evidenced by the localization of cell-cell transmembrane protein N-cadherin (Supplementary Fig. 10). The trend was also seen in gene expression analysis, especially in serum starvation where hCEnC markers were slightly increased compared to En medium (Supplementary Fig. 11). Interestingly, the expression of AQP1 was markedly elevated, that could be potentially due to the hPSC-CEnCs’ response to prevent edema^58^, which starvation might hypothetically induce through the death of other cell populations. Additionally, it has been demonstrated that AQP1 is upregulated in migrating hCEnC^59^, a response that could be triggered as the cells attempt to cover newly formed empty spaces. The elevated expression of LUM suggests that neural crest-derived hPSC-CK-like cells could also benefit from the starvation protocol. In addition, the TFAP2A expression was increased in hPSC-CEnCs starvation cultures. The NCC marker AP2α, produced by the TFAP2A gene, has been identified as characteristic of hPSC-CEnCs produced using our previously developed differentiation protocol^26^ where a relative increase in TFAP2A expression indicates more enriched hPSC-CEnC population. However, as a conclusion from these starvation tests, we need to emphasize that, the variability between batches of cells used for starvation, highlights the importance for further development of the method. Also, the most accurate time point to stop the starvation phase needs to be optimized further in addition to the further development of culture conditions after starvation to prevent reaccumulating mesenchymal-like cell clusters (Supplementary Fig. 12). However, the gained results provided potential purification strategy that can be developed further. In conclusion, our findings provide potential solutions to enrich both the hPSC-CK-like cells and the hPSC-CEnC using the same signaling pathway induction method for hPSC differentiation.

## Electronic supplementary material

Below is the link to the electronic supplementary material.

Supplementary Material 1

## Data Availability

All data generated or analysed during this study are included in this published article (and its Supplementary Information files).
