# Supplementary material for: Co-differentiation and enrichment of corneal endothelial cells and keratocytes from human pluripotent stem cells
Source: Sci Rep. 2025 May 28;15:18750. doi: 10.1038/s41598-025-03509-3 (PMC12120075; doi:10.1038/s41598-025-03509-3)
Supplement: Supplementary file 1 — Supplementary Material 1 [file 41598_2025_3509_MOESM1_ESM.pdf]

Supplementary material

1.1. Supplementary methods

Supplementary method 1. Reverse transcription (RT)-PCR

For RT-PCR, forward and reverse primers (Supplementary Table 2) were designed (OligoPerfect Designer, Invitrogen and Primer-BLAST 29) and used with DNA polymerase (DreamTaq DNA Polymerase, Cat# EP0702, Thermo Scientific) and dNTPs (dNTP Mix, Cat# R0241, Thermo Scientific) for gene amplification by PCR. The final PCR product was analyzed by agarose gel electrophoresis imaging (Gel Doc XR+, Bio-Rad with Image Lab Software) and comparing the PCR product size with a DNA ladder (GeneRuler 50 bp DNA Ladder, Cat# SM0371, Thermo Scientific).

Supplementary figures

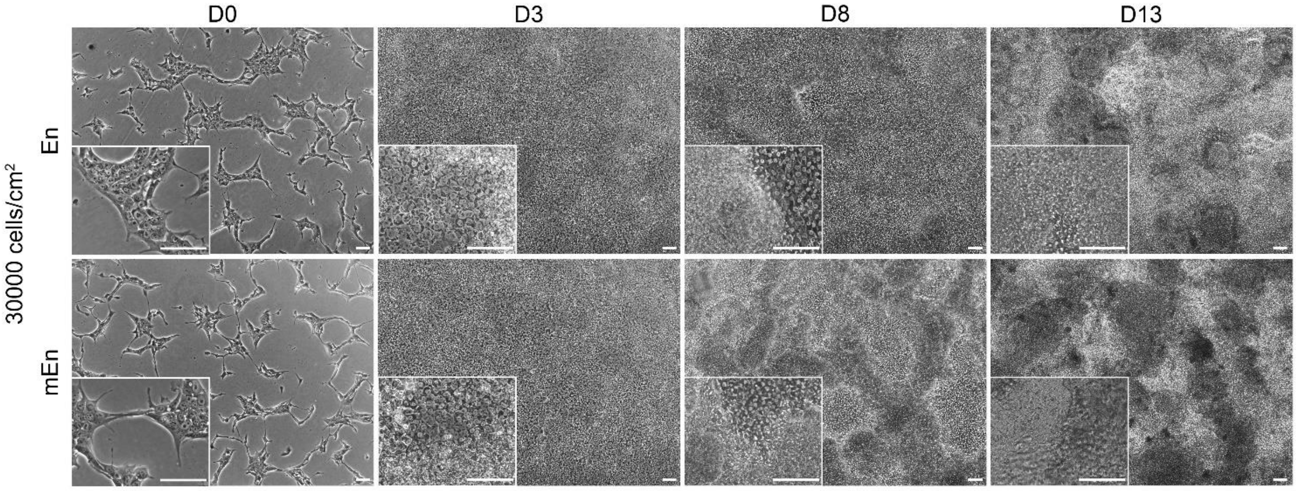

Supplementary Figure 1. Differentiation of hESC into CK-like cells with high seeding densities. Phase contrast image panel shows the hESC seeded at a density of 30000 cells/cm^2. Cells were confluent by day (D) 3 with both En and mEn protocols. Cell clusters began to develop by D8 and increase by D13. Individual cell morphologies were however not very clear in the cell clusters and only few areas showed polygonal CEnC. Data shown is the representative images of the hESC differentiated to CK-like cells (n=3). Objective magnification: 4x and 10x. Scale bar: 100 µm.

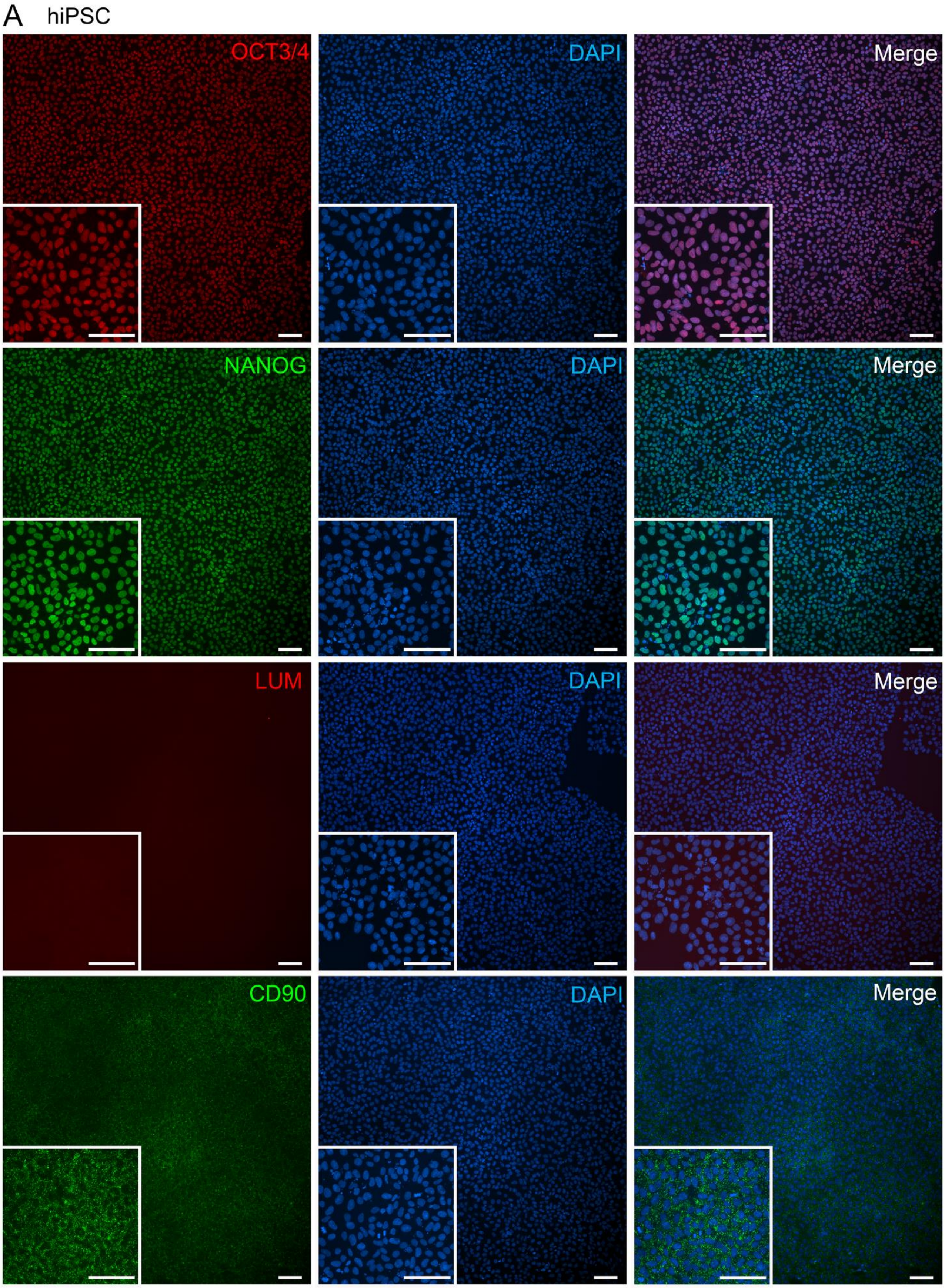
A
hiPSC
OCT3/4
DAPI
Merge
NANOG
DAPI
Merge
LUM
DAPI
Merge
CD90
DAPI
Merge

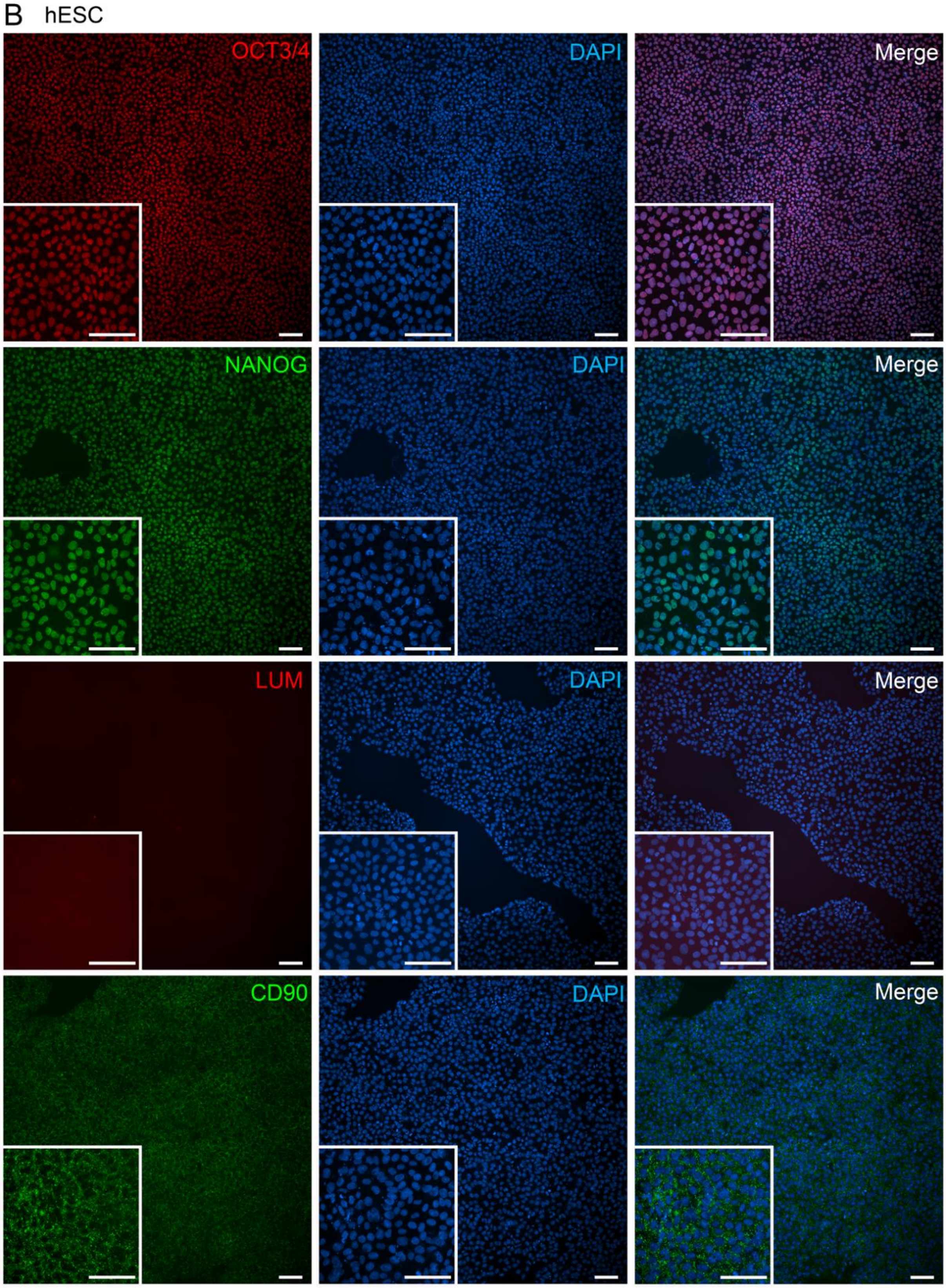

Supplementary Figure 2. Expression of CK markers in undifferentiated hPSC. Expression of OCT3/4 and Nanog in undifferentiated cells indicating the pluripotent stem cell characteristics in hiPSC (A) and hESC (B). Both cell types were CD90 positive and lumican (LUM) negative. Data shown is representative images of both hiPSC (WT001.TAU.bB) and hESC (n=3). Objective magnification: 10x and 20x. Scale bar: 100 µm.

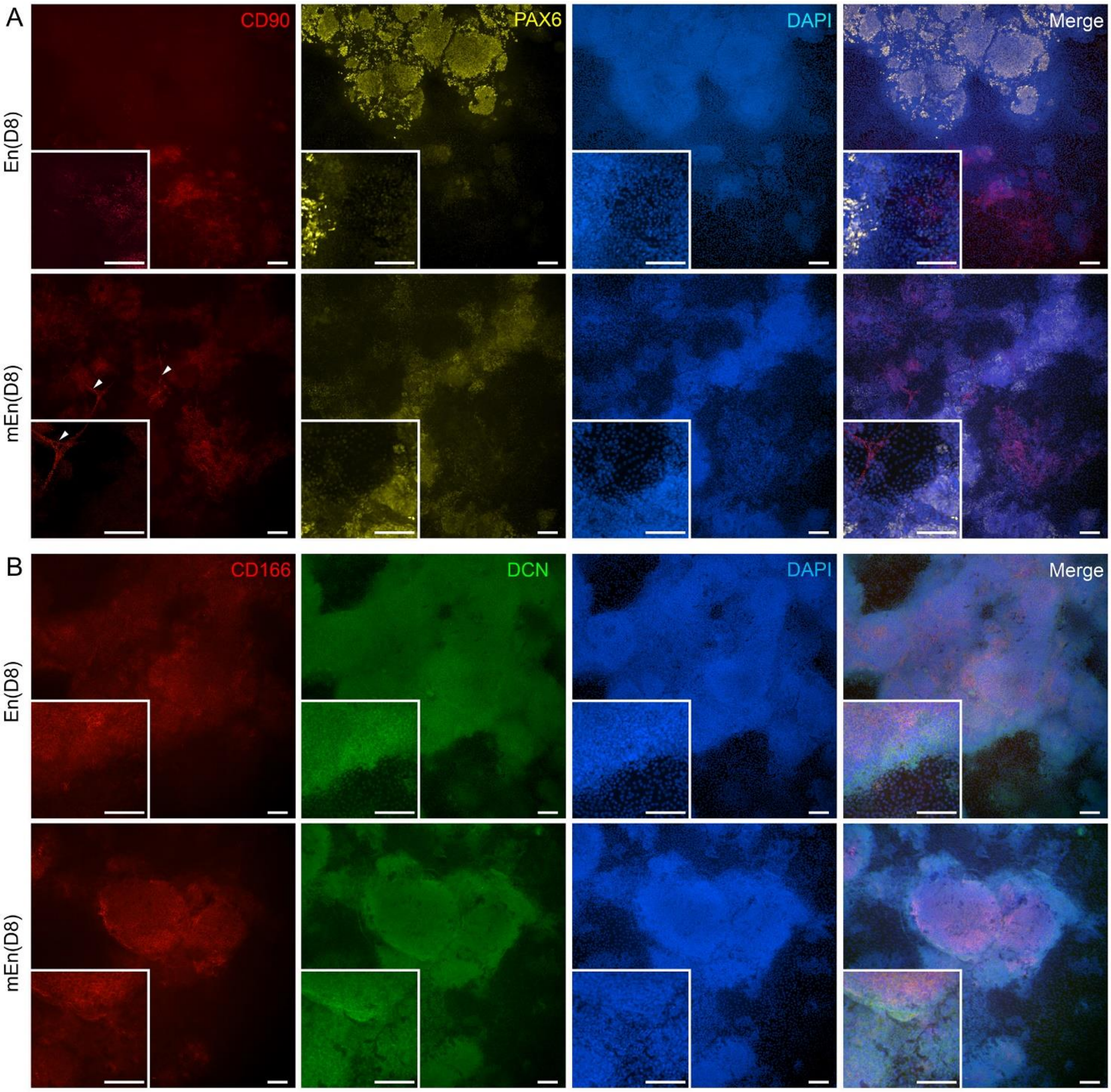
A
CD90
PAX6
DAPI
Merge
En(D8)
mEn(D8)
B
CD166
DCN
DAPI
Merge
En(D8)
mEn(D8)

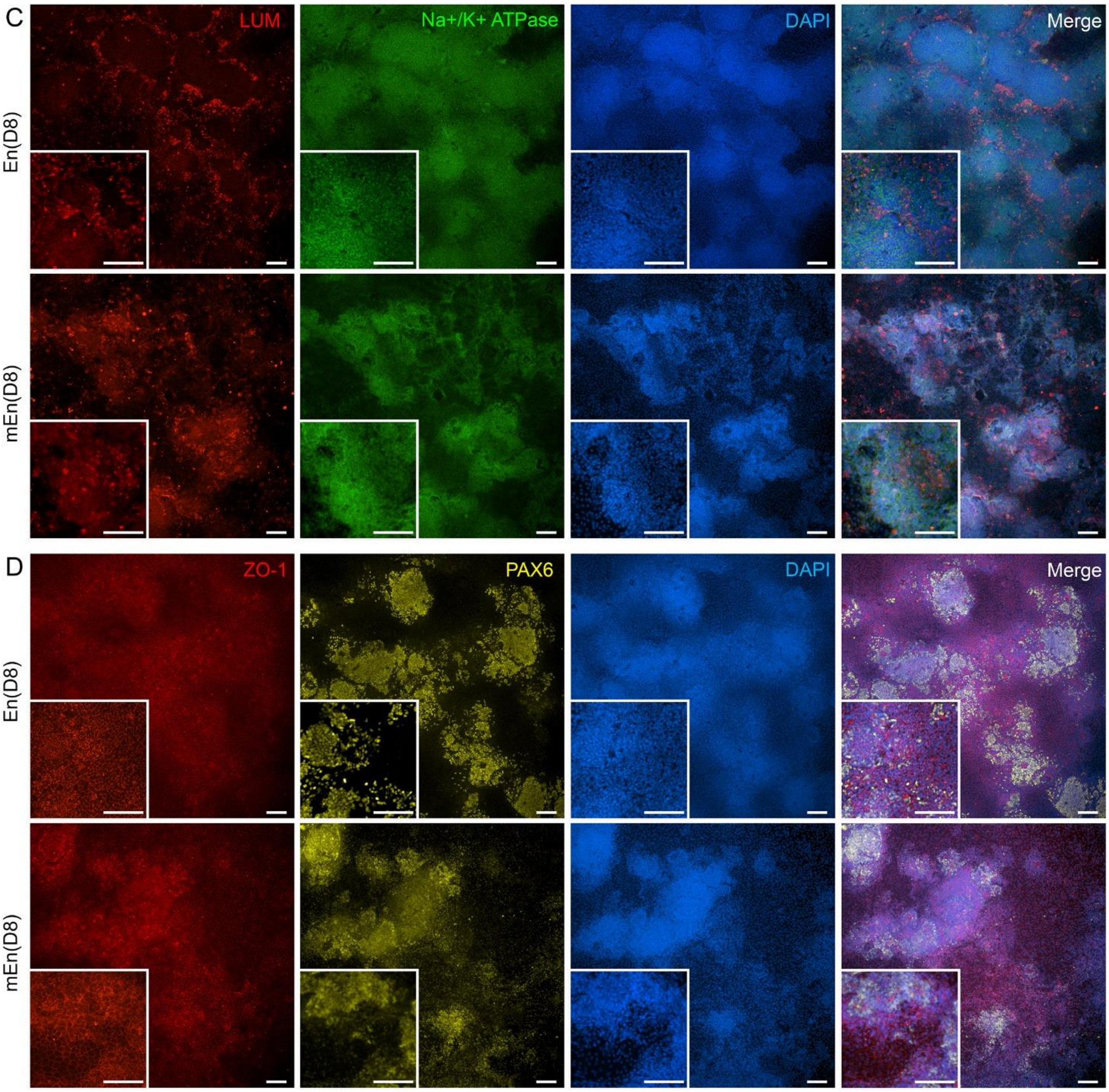

C
LUM
Na+/K+ ATPase
DAPI
Merge
En(D8)
mEn(D8)
D
ZO-1
PAX6
DAPI
Merge
En(D8)
mEn(D8)

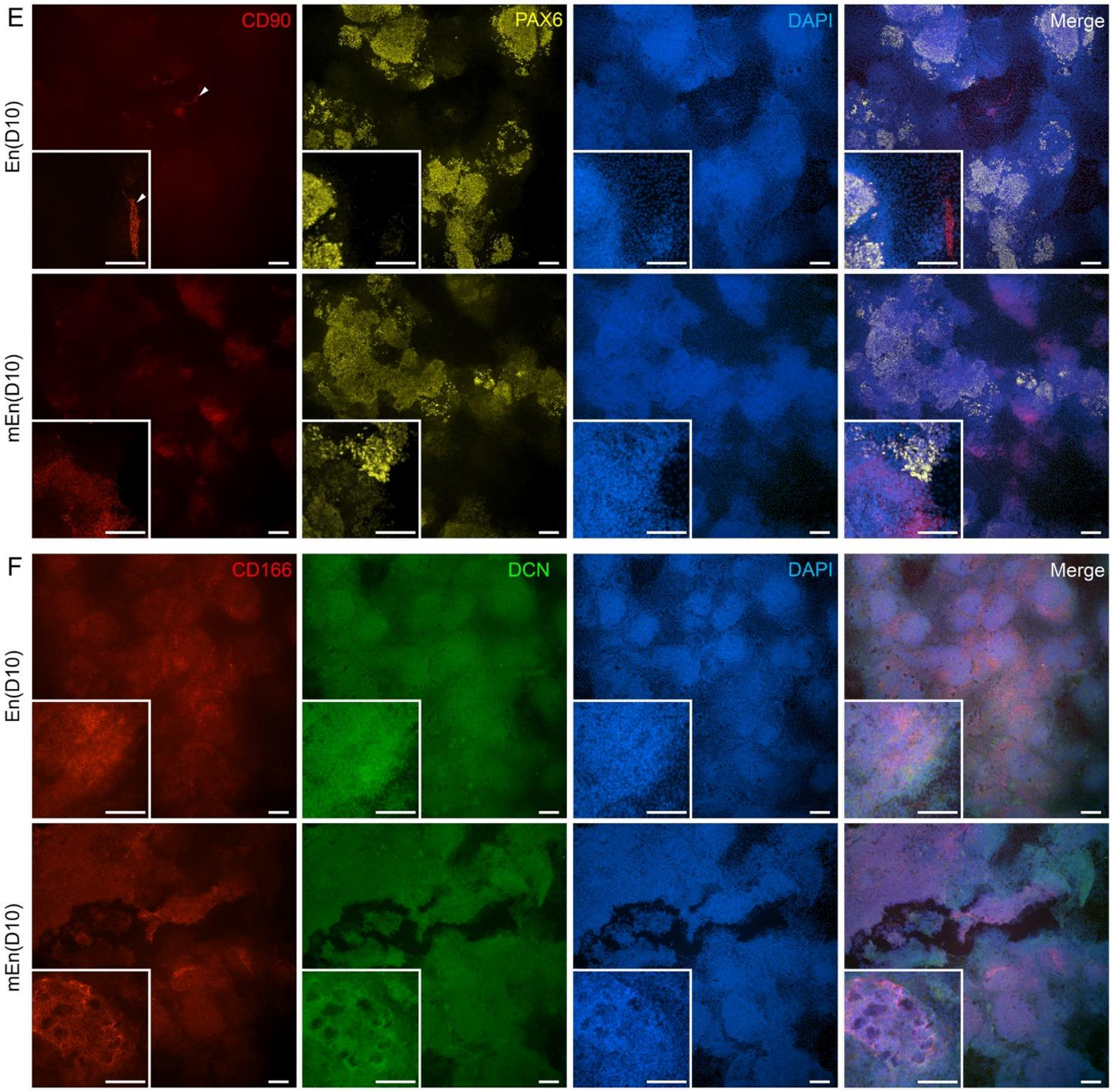
E
CD90
PAX6
DAPI
Merge
En(D10)
mEn(D10)
F
CD166
DCN
DAPI
Merge
En(D10)
mEn(D10)

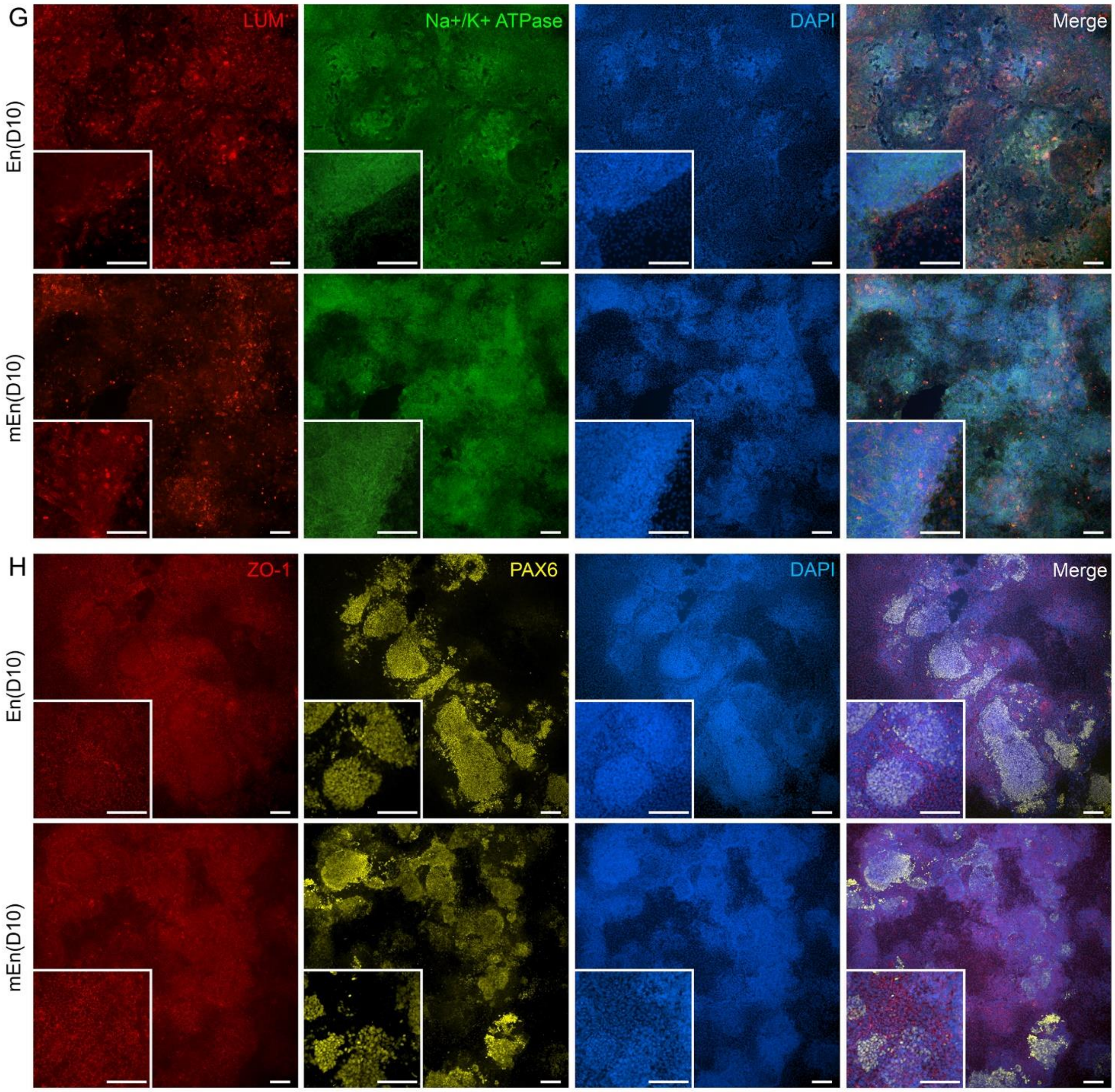

Supplementary Figure 3. Expression of CK and CEnC markers in hPSC differentiated CK-like cells. IF shows the expression of CD90 and PAX6 (A (D8) and E (D10)), CD166 and decorin (DCN) (B (D8) and F (D10)), lumican (LUM) and Na^+/K^+-ATPase (C (D8) and G (D10)) and ZO-1 and PAX6 (D (D8) and H (D10)) in both Endothelial (En) and modified endothelial protocols (mEn) after differentiation. Abundant expression of the proteoglycans (LUM and DCN) was observed. Additionally, CD90 was expressed in cells with fibroblastic morphology and ZO-1 have outlined the polygonal morphology of the cells indicating CEnC. Data shown is representative images of the hiPSC (WT001.TAU.bB) differentiated to CK-like cells (n=4) with two technical replicates in each. White arrow heads indicate cells with fibroblast-like morphology. Objective magnification: 10x and 20x. Scale bar: 100 µm.

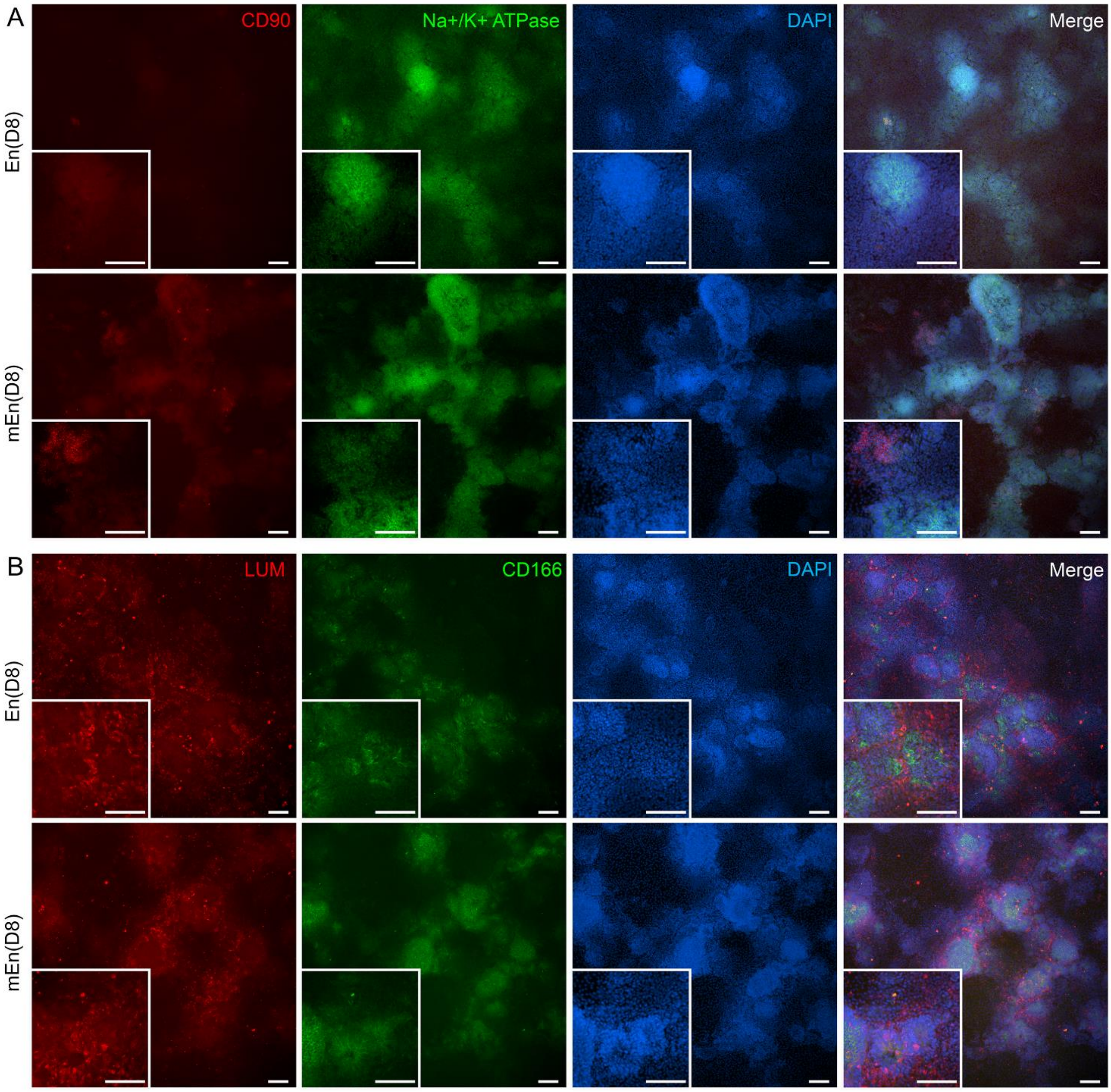
A
CD90
Na+/K+ ATPase
DAPI
Merge
En(D8)
mEn(D8)
B
LUM
CD166
DAPI
Merge
En(D8)
mEn(D8)

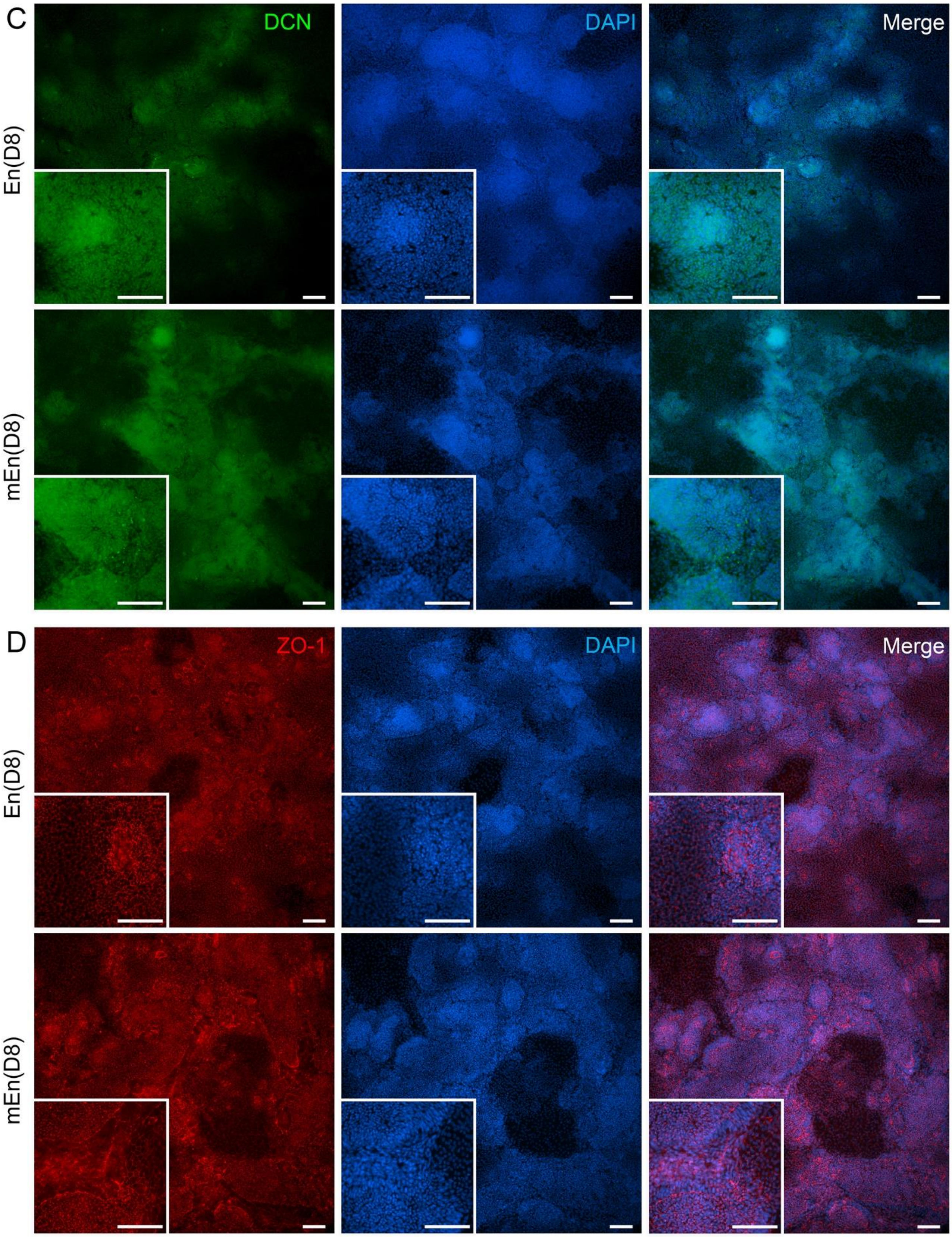
C
DCN
DAPI
Merge
En(D8)
mEn(D8)
D
ZO-1
DAPI
Merge
En(D8)
mEn(D8)

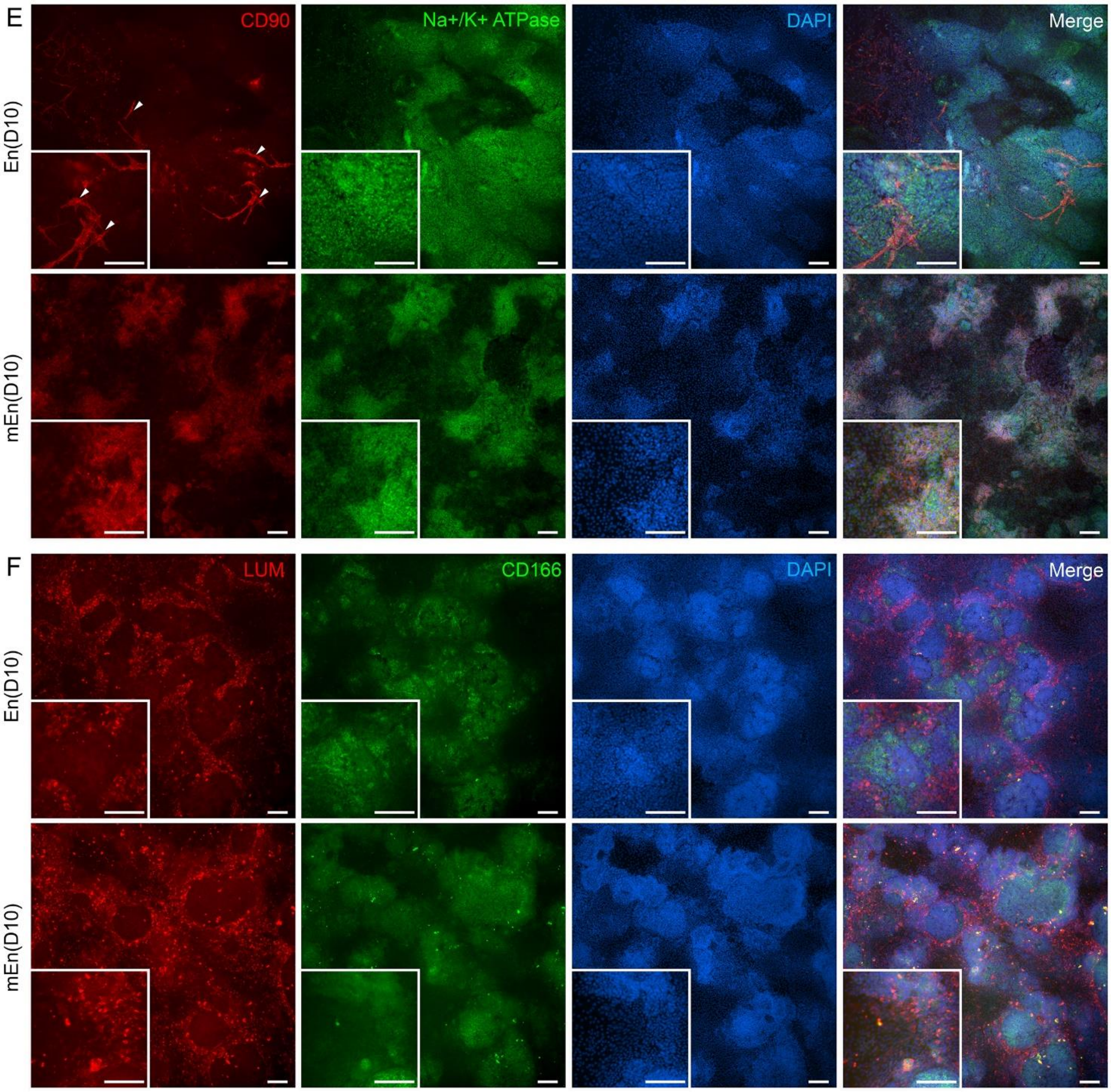
E
CD90
Na+/K+ ATPase
DAPI
Merge
En(D10)
mEn(D10)
F
LUM
CD166
DAPI
Merge
En(D10)
mEn(D10)

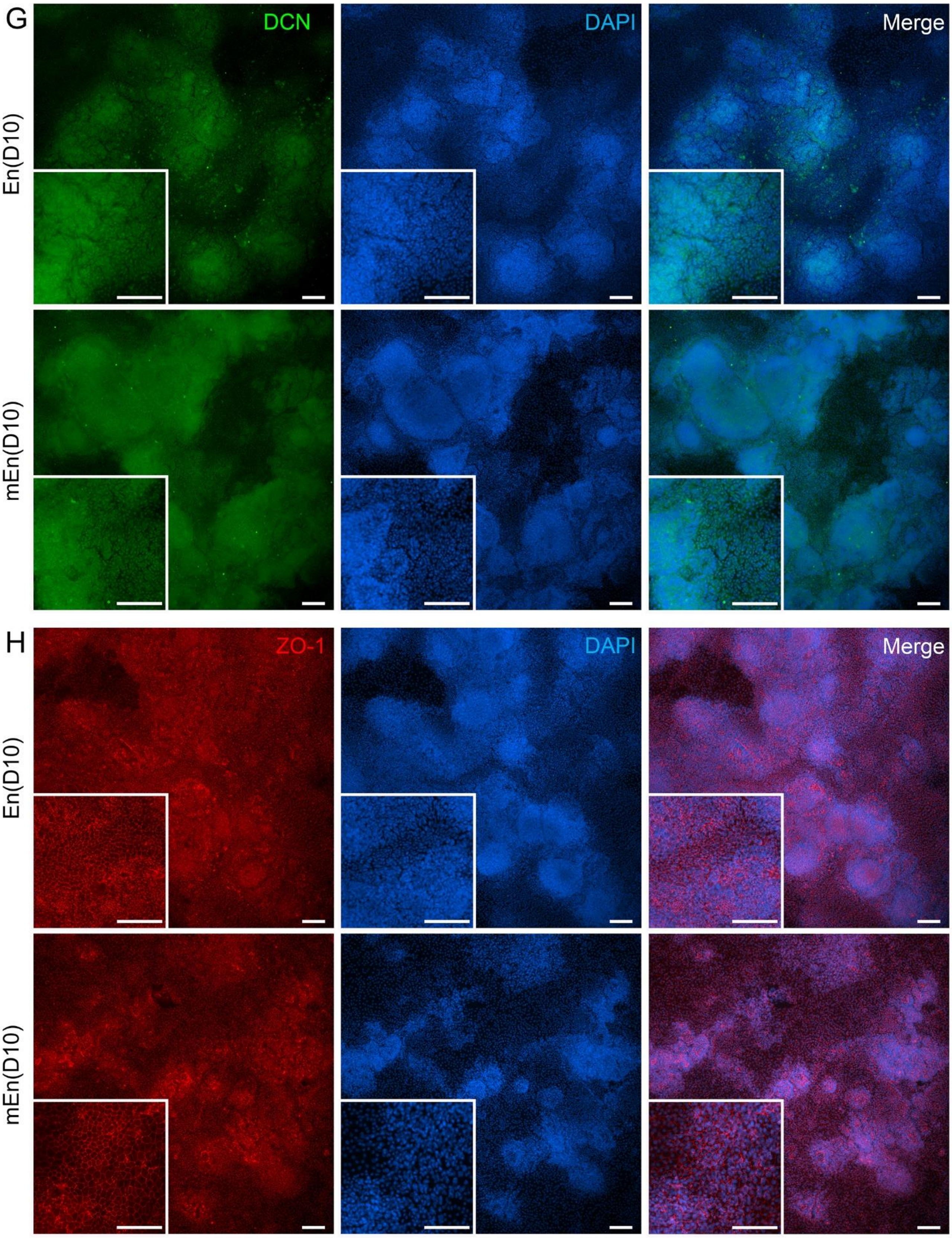

G
DCN
DAPI
Merge
En(D10)
mEn(D10)
H
ZO-1
DAPI
Merge
En(D10)
mEn(D10)

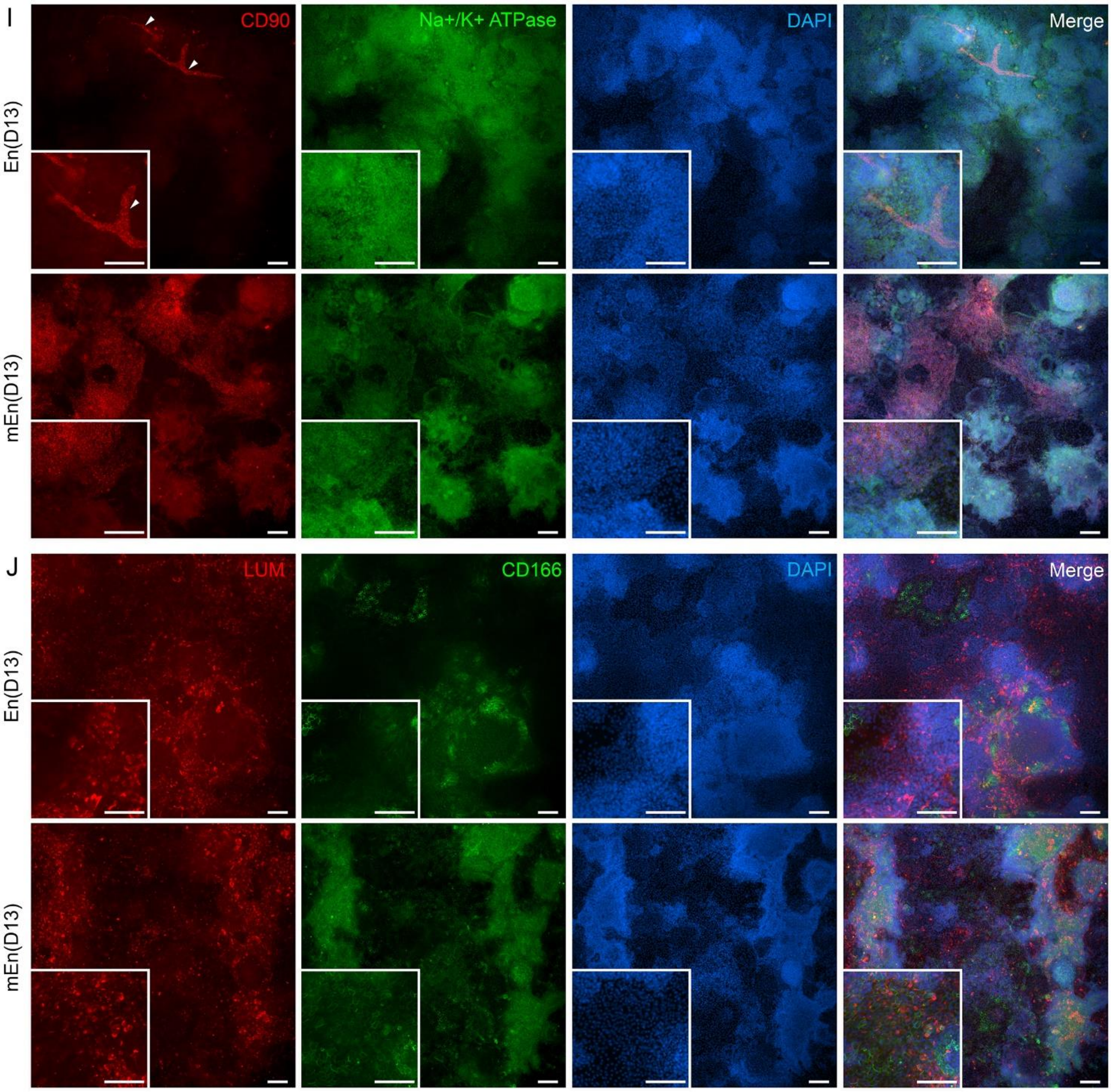

I
CD90
Na+/K+ ATPase
DAPI
Merge
En(D13)
mEn(D13)
J
LUM
CD166
DAPI
Merge
En(D13)
mEn(D13)

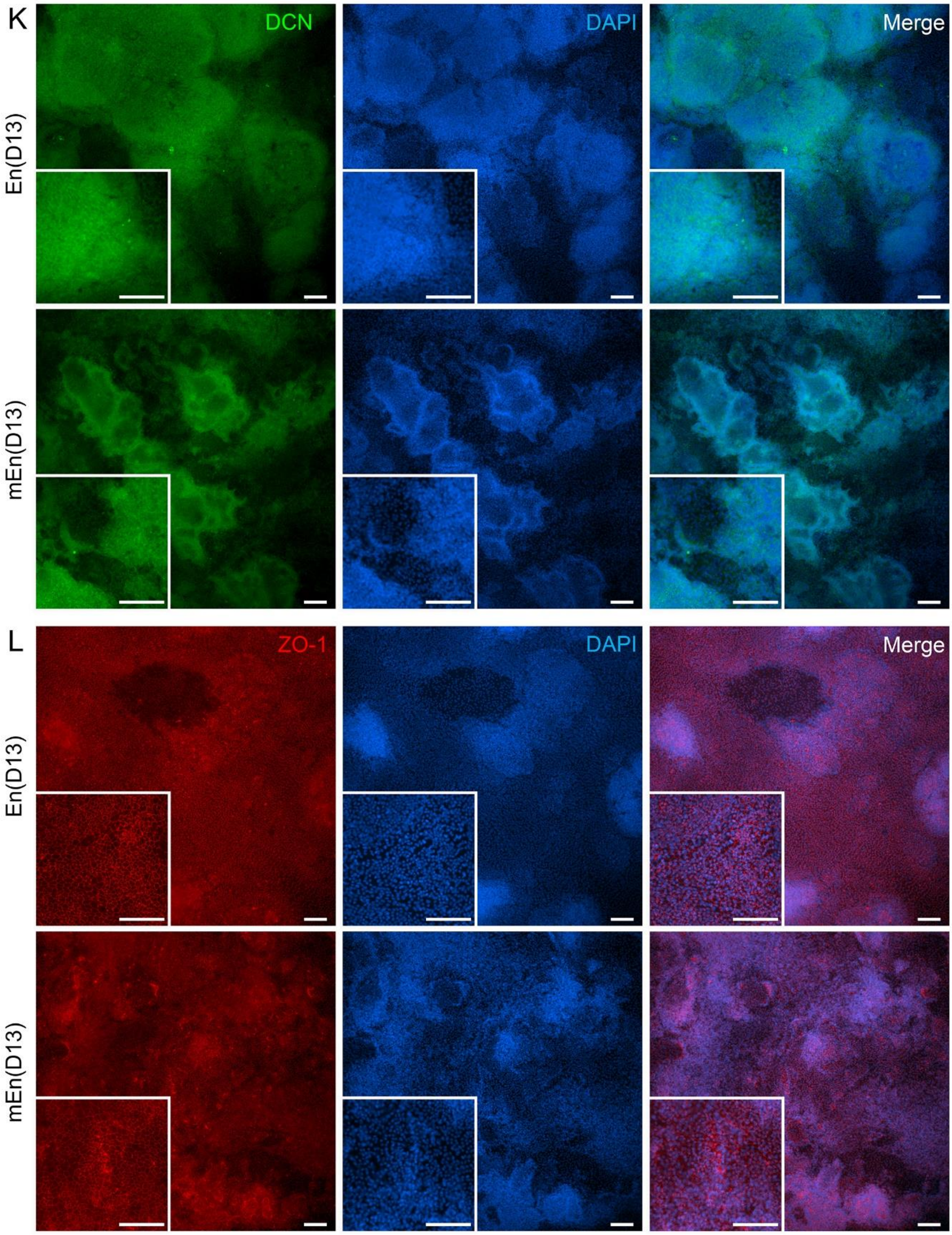

Supplementary Figure 4. Expression of CK and CEnC markers in hESC differentiated CK-like cells. IF shows the expression of CD90 and Na+/K+-ATPase (A (D8), E (D10) and I (D13)), lumican (LUM) and CD166 (B (D8), F (D10) and J (D13)), decorin (DCN) (C (D8), G (D10) and K (D13)), and ZO-1 (D (D8), H (D10) and L (D13)) in both En and mEn protocols after differentiation. Abundant expression of the proteoglycans (LUM and DCN) was observed. Additionally, CD90 was expressed in cells with fibroblastic morphology and ZO-1 have outlined the polygonal morphology of the cells indicating CEnC. Data shown is representative images of the hESC differentiated to CK-like cells (n=3). White arrow heads indicate cells with fibroblast-like morphology. Objective magnification: 10x and 20x. Scale bar: 100 µm.

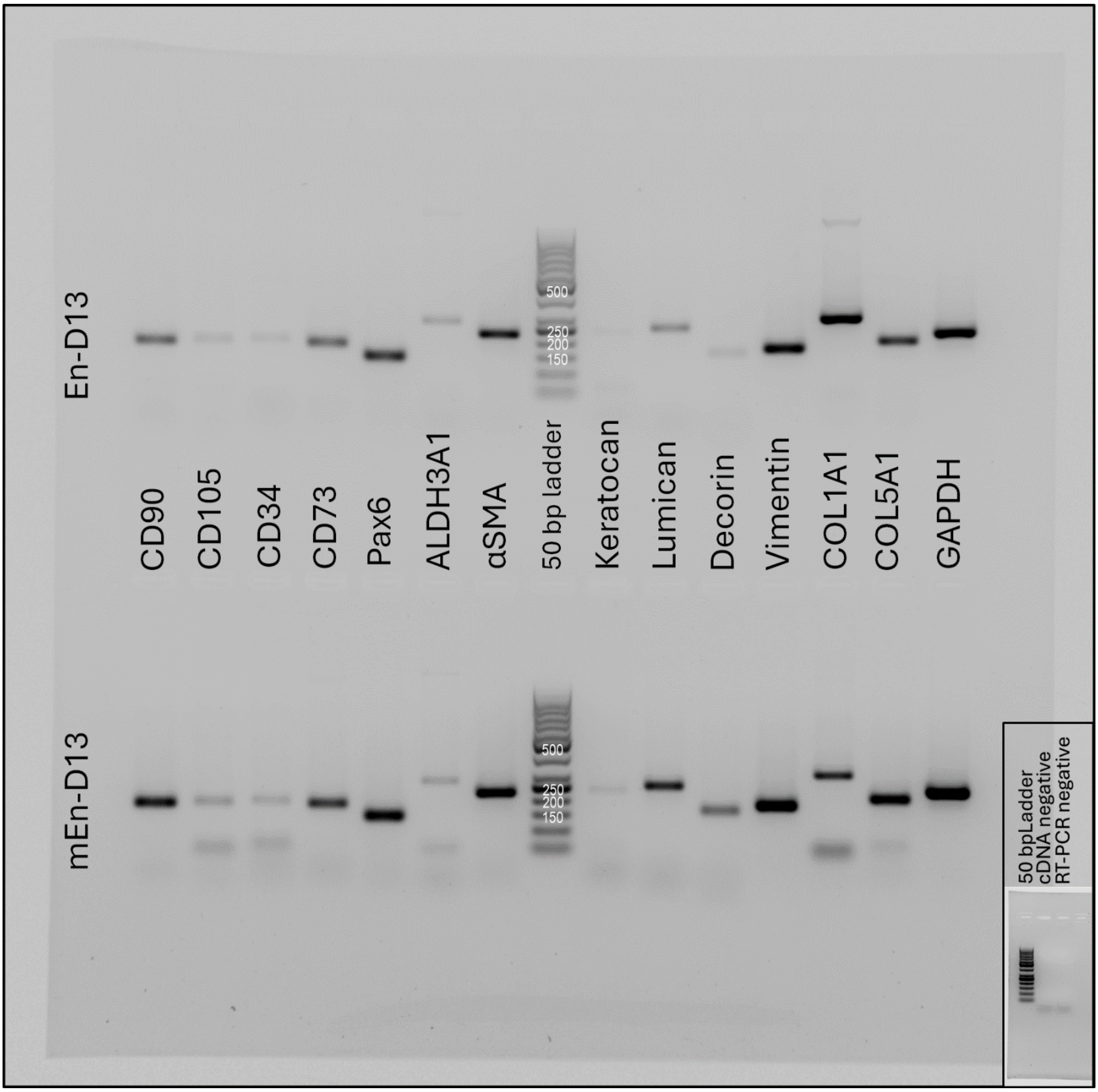

Supplementary Figure 5. Screening of corneal stromal cell related genes in hPSC-CK by semi-quantitative gene expression. Representative image showing the corneal stromal/CK-specific genes expressed in hiPSC-CK-like cells at day (D)13 in both endothelial (En) and modified endothelial (mEn) protocols. Image shown here is a single agarose gel (15 x 15 cm) with two rows of wells (15 wells per row) containing PCR products and an inset image from another gel showing negative controls. Picture was obtained with a gel document imager (Gel Doc XR+, Bio-Rad with Image Lab Software) at 0.01 second exposure. White markings represent the band size of the reference ladder used for identifying the specific size of the PCR products in the agarose gel. Inset image is obtained at 0.1 second exposure (with cropped empty wells to right) showing no band in the negative control of cDNA preparation and RT-PCR samples. Data shown is representative image of the hiPSC (WT001.TAU.bB) differentiated to CK-like cells (n=3).

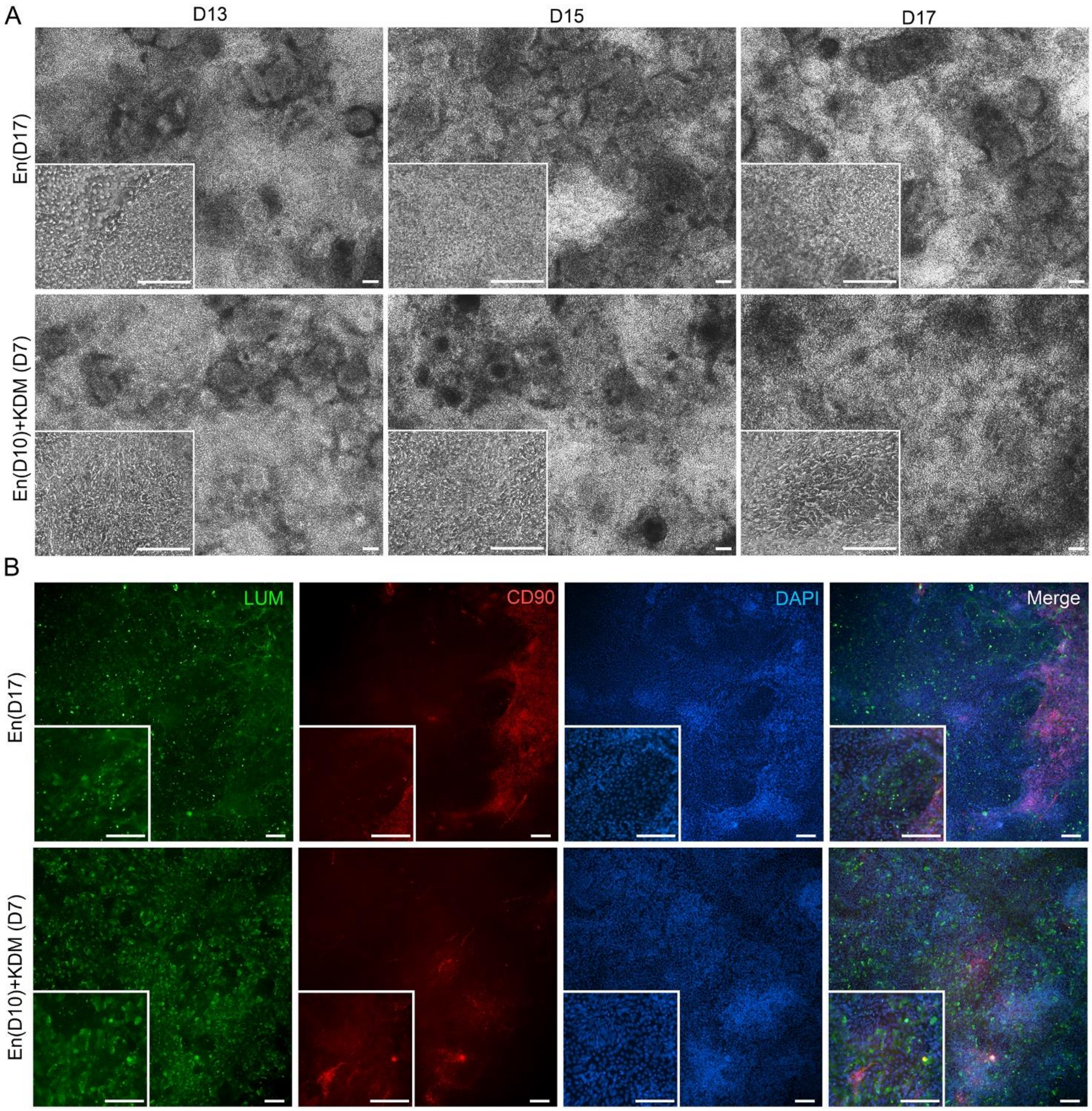

Supplementary Figure 6. Differentiation cultures of hPSC-CK. En (D17) represents continuous cultures of hPSC-CK until day (D) 17. En (D10) + KDM (D7) represents En cultures until D10 and then switched to keratocyte differentiation medium (KDM) to culture for 7 more days. A) Phase contrast images of the hPSC-CK cultures demonstrates fibroblast-like morphology on D17 in KDM culture (inset image). B) IF shows the increased expression of lumican (LUM) and decreased CD90 in En-D10 + KDM-D7 compared to En-D17 indicating the influence of KDM on hPSC-CK characteristics. Data is the representative images of hiPSC (WT001.TAU.bB) differentiated to CK-like cells (n=2). Objective magnification: A) 4x and 10x. B) 10x and 20x. Scale bar: 100 µm.

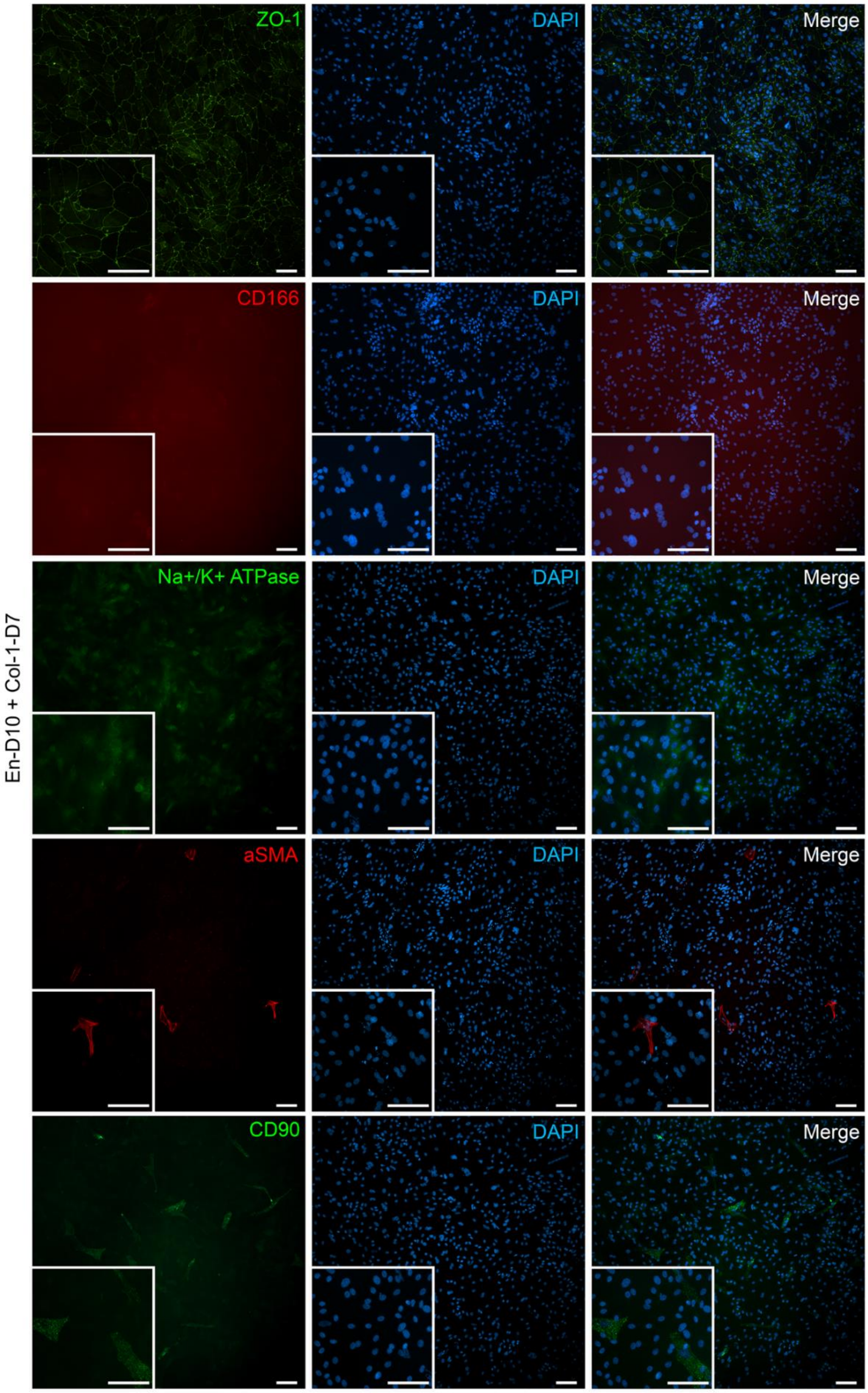

Supplementary Figure 7. Expression of Corneal stromal cell (CSC) or CEnC markers in enriched hPSC-CK. IF of hPSC-CK-like cells enriched on collagen-1 coated plates had minimal ZO1 expression (green) and Na^+/K^+-ATPase (green). Moreover, no CD166 expression was observed indicating the absence of CEnC phenotype. CSC markers CD90 (green) and alpha SMA (aSMA; red) was observed. Data is the representative images of hiPSC (WT001.TAU.bB) differentiated to CK-like cells (n=3). Objective magnification: 10x and 20x. Scale bar: 100 µm.

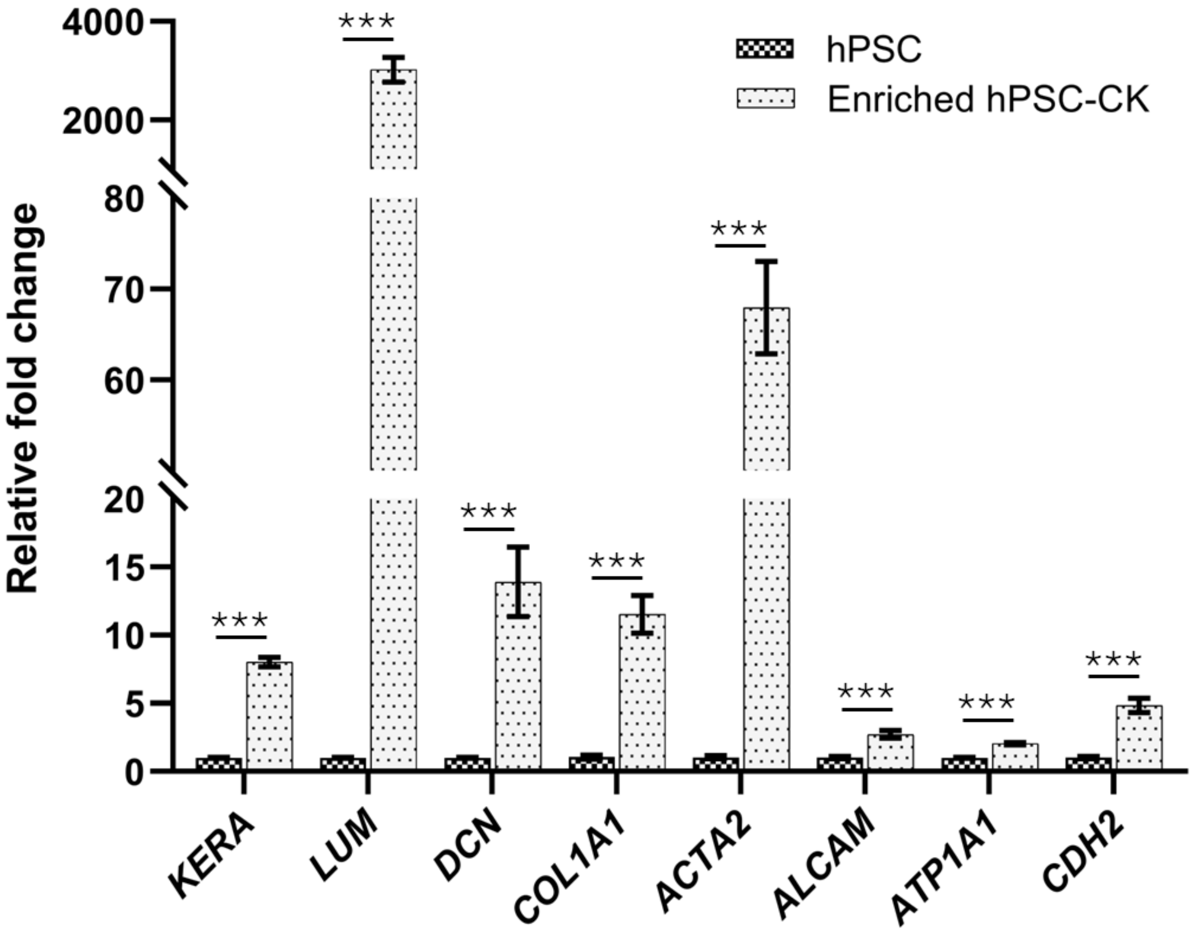

Supplementary Figure 8. Gene expression analyses of the enriched hPSC-CK-like cells compared to hPSC. Gene expression analysis of the enriched hPSC-CK-like cells cultured on hCOL1 plates showed significant increase in expression of all the genes. The relative fold changes of the analyzed genes (KERA: 8.04±0.35; LUM: 3020.85±250; DCN: 13.91±2.55; COL1A1: 11.54±1.38; ACTA2: 67.95±5.09; ALCAM: 2.72±0.28; ATP1A1: 2.04±0.08; CDH2: 4.85±0.53; PAX6: 8.04±0.35;) were highly significant ($p \leq 0.001$) when compared to their expression in undifferentiated hPSC. Mann-Whitney U test was performed to compare the ranks among the groups of a gene. Significant relative fold change was indicated as *** - $p \leq 0.001$. Data is represented as the mean relative fold changes of the gene with standard error in hiPSC (WT001.TAU.bB) differentiated to CK-like cells (n=3).

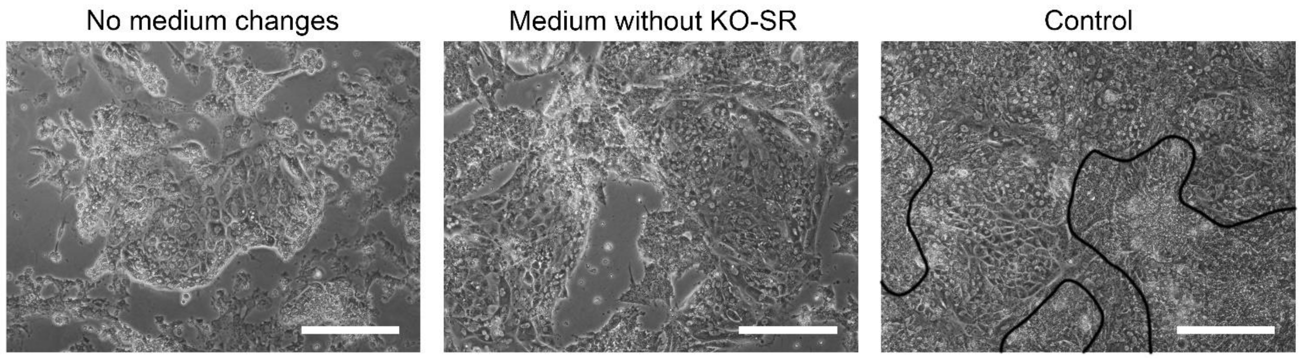

Supplementary Figure 9. Starvation methods without medium changes and medium without KO-SR compared to control. No major differences were observed between the two starvation methods, both reduced the mesenchymal-like cell structures which can be seen in control culture, where medium was changed normally. Black marker profiles the mesenchymal-like structures. Data shown is representative images of the hESC differentiated to CEnC-like cells (n=3). Objective magnification: 10x. Scale bars 200 µm.

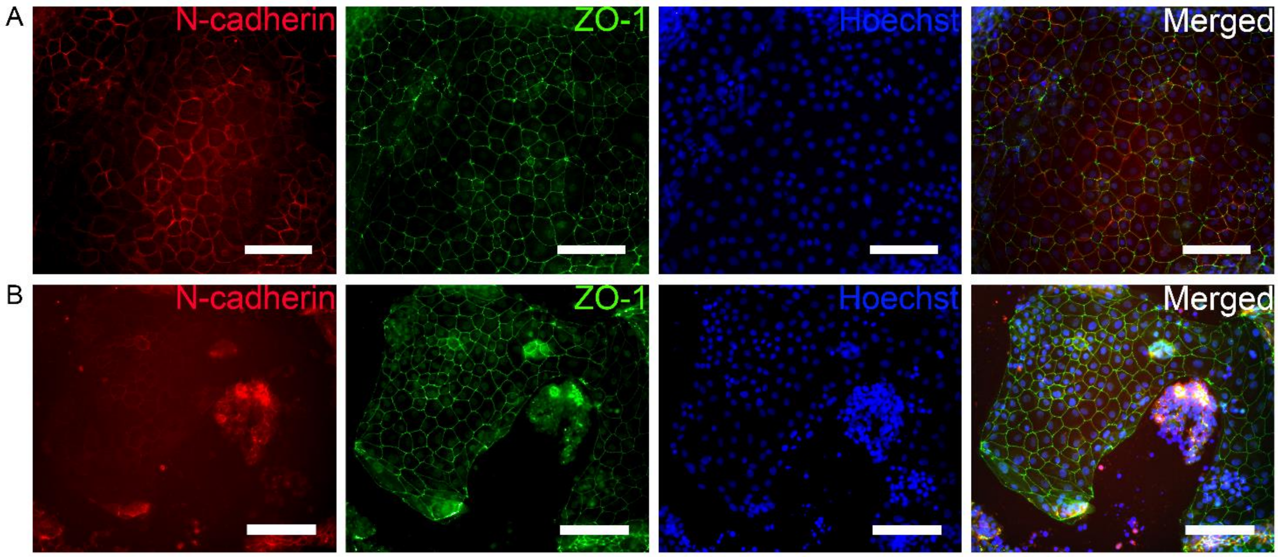

Supplementary Figure 10. Structural integrity of hPSC-CEnCs after starvation. Immunofluorescence images of the cells after 6 days of starvation. ZO-1 (green) is localized in tight junctions and N-cadherin (red) in lateral membrane. A) Starvation without medium changes, hESC line Regea08/017 B) Starvation using En medium without KO-SR, hiPSC line AICS-0016-184. Number of biological replicates n=4. Objective magnification 20x. Scale bars 100 µm.

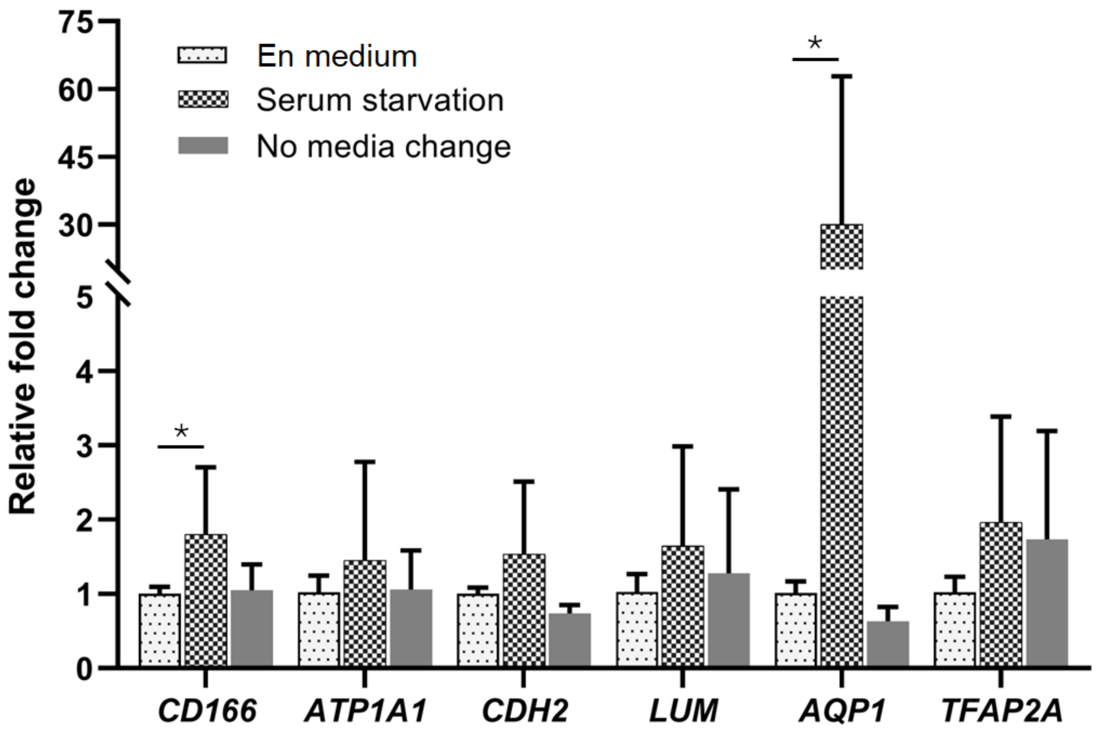

Supplementary Figure 11. Gene expression analyses of hPSC-CEnCs under serum starvation and no media change methods were compared to normal En medium changes. The expression of hCEnC markers CD166, ATP1A1, CDH2, and AQP1 was higher in the serum starvation method compared to no media changes, indicating a purification of the cell population. However, only CD166 and AQP1 showed significant differences. LUM exhibited a slight increase in the starvation method, and TFAP2A also increased, suggesting an elevated relative amount of hPSC-CEnCs in the culture. Kruskal-Wallis test was used to compare multiple means with Dunn's correction. Significant relative fold changes were indicated as * - $p \leq 0.05$. Data is represented as the mean relative fold changes of the gene with standard error (n=3). Data collected from three biological replicates (n=3) using two replicates from Regea08/017 cell lines and one from AICS-0016-184 cell line.

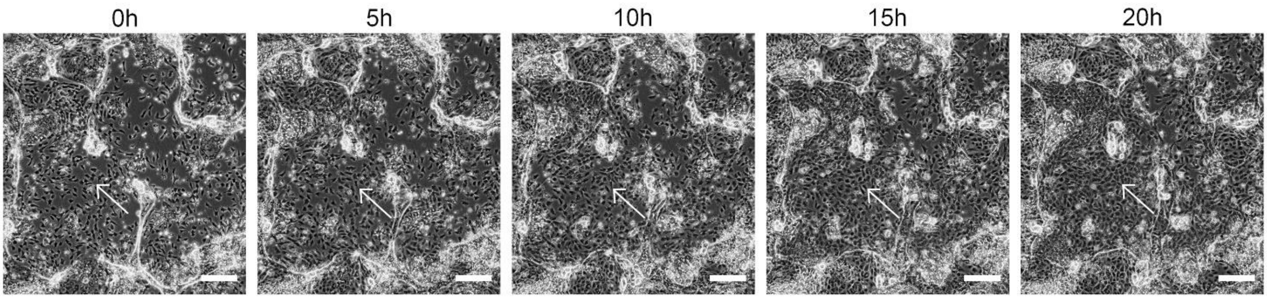

Supplementary Figure 12. Timelapse images of post starvation mesenchymal growth in thawed hPSC-CEnC culture. Cells were first cultured 3 days in normal En medium and then being 5 days in medium without KO-SR (serum starvation). Finally, the cells were cultured for two days in normal En medium. The first 20 hours of this period are shown in the time-lapse imaging, with arrows indicating one of the areas where mesenchymal-like cells are proliferating. Data shown is representative images of the hESC differentiated to CEnC-like cells (n=3). Objective magnification: 10x. Scale bars 200 μm.

1.3. Supplementary Tables

Supplementary Table 1. Protein markers to define cell types

hPSC	OCT 3/4<br>Nanog
Eye field marker	PAX6
Neural crest cells	AP2a
Corneal stromal stem cells	CD90
hPSC-CK	Lumican (LUM)<br>Decorin (DCN)<br>Vimentin
hPSC-CEnC	CD166<br>N-Cadherin<br>Na+/K+-ATPase<br>ZO-1

Supplementary Table 2. List of antibodies used for immunofluorescence

**Antibody**	**Dilution**	**Catalog/Manufacturer/RRID (Research Resource Identifiers)**
Zonula occludens-1 (ZO-1)	1:400	Cat# 61-7300, Thermo Fisher Scientific, RRID:AB_2533938
Sodium potassium ATPase (Na+/K+-ATPase)	1:200	Cat# ab7671, Abcam, RRID:AB_306023
Sodium potassium ATPase (Na+/K+-ATPase)	1:300	Cat# 55187-1-AP , Proteintech, RRID:AB_10859261
CD166	1:400	Cat# 559260, BD Biosciences, RRID:AB_397209
Ki67	1:400	Cat# AB9260, Millipore, RRID:AB_2142366
Activating enhancer binding protein 2 alpha (AP2α)	1:200	Cat# sc-12726, Santa Cruz Biotechnology, RRID:AB_667767
Lumican (LUM)	1:300	Cat# AF2846, R and D Systems, RRID:AB_2139484
PAX6	1:300	Cat# HPA030775, Sigma-Aldrich, , RRID:AB_10601243
THYmocyte differentiation antigen 1 (Thy-1 or CD90)	1:100	Cat# sc-53456, Santa Cruz Biotechnology, RRID:AB_630308
Decorin (DCN)	1:100	Cat# PA5-13538, Thermo Fisher Scientific, RRID:AB_2090260
Decorin (DCN)	1:200	Cat# 14667-1-AP, Thermo Fisher Scientific, RRID:AB_2090265
Vimentin (VIM)	1:300	Cat# AB1620, Millipore, RRID:AB_90774
Nanog	1:300	Cat# AF1997, R and D Systems, RRID:AB_355097

N-cadherin	1:300	Cat# 33-3900, Thermo Fisher Scientific, RRID:AB_2313779
Octamer binding transcription factor 3/4 (OCT3/4)	1:300	Cat# AF1759, R and D Systems, RRID:AB_354975
Anti-Mouse IgG Alexa Fluor™ 488	1:800	Cat# A-21202, Thermo Fisher Scientific, RRID:AB_141607
Anti-Mouse IgG Alexa Fluor™ 568	1:800	Cat# A10037, Thermo Fisher Scientific, RRID:AB_11180865
Anti-Rabbit IgG Alexa Fluor™ 568	1:800	Cat# A10042, Thermo Fisher Scientific, RRID:AB_2534017
Anti-Rabbit IgG Alexa Fluor™ 647	1:800	Cat# A-31573, Thermo Fisher Scientific, RRID:AB_2536183
Anti-Goat IgG Alexa Fluor™ 568	1:800	Cat# A-11057, Thermo Fisher Scientific, RRID:AB_2534104
Anti-Goat IgG Alexa Fluor® 647	1:800	Cat# ab150131, Abcam, RRID:AB_2732857

Supplementary Table 3. List of primers used for reverse transcription (RT)-PCR and RT-qPCR

**Primers for RT-PCR**		
**S. No.**	**Primer sequence**	**NCBI Ref Seq_Species_Primer name**
**1**	ATCAGGAGTTCCAGTGCTGC	BC065559.1_Hu_CD90 (Thy-1)_FWD
**2**	TGGCTTCCCTCTTCACGAAC	BC065559.1_Hu_CD90 (Thy-1)_REV
**3**	GTGTGAACTCACCCTGGGAG	BC014271.2_Hu_CD105 (Endoglin)_FWD
**4**	AGCCATATCCCAGACCCACT	BC014271.2_Hu_CD105 (Endoglin)_REV
**5**	CAGCAAGACAACACGTGGTG	M81104.1_Hu_CD34_FWD
**6**	CCCCAAGAACAGCCTCTGAG	M81104.1_Hu_CD34_REV
**7**	TATCCGGTCGCCCATTGATG	BC065937.1_Hu_CD73_FWD
**8**	CTGCAGGAACTCTCCAGTGG	BC065937.1_Hu_CD73_REV
**9**	AGAACAGTCACAGCGGAGTG	AY047583.1_Hu_PAX6_FWD
**10**	GACACCTGCAGAATTCGGGA	AY047583.1_Hu_PAX6_REV
**11**	GCAGGACGAGCTCTACATCC	BC008892.2_Hu_ALDH3A1_FWD
**12**	GGTCGAACCTCTCCTTGAGC	BC008892.2_Hu_ALDH3A1_REV
**13**	ACTGCCTTGGTGTGTGACAA	BC093052.1_Hu_aSMA_FWD
**14**	TCCCAGTTGGTGATGATGCC	BC093052.1_Hu_aSMA_REV
**15**	TGTTTCTGCCCACCCAGTTT	AF205403.1_Hu_KERA_FWD
**16**	TTCTTCAGCTGGCTTAGGGC	AF205403.1_Hu_KERA_REV
**17**	CCAATGGTGCCTCCTGGAAT	U18728.1_Hu_LUMI_FWD
**18**	AGCTGCAGATCCTCCAGAGA	U18728.1_Hu_LUMI_REV
**19**	CTTGCACAAGTTTCCTGGGC	BC005322.1_Hu_Decorin_FWD

**20**	CGAAGATGGCATTGACAGCG	BC005322.1_Hu_Decorin_REV
**21**	GGACCAGCTAACCAACGACA	BC066956.1_Hu_Vimentin_FWD
**22**	AAGGTCAAGACGTGCCAGAG	BC066956.1_Hu_Vimentin_REV
**23**	AGGAGAGAGAGGCTTCCCTG	JQ236861.1_Hu_COL1A1_FWD
**24**	ACGATCACCACTCTTGCCAG	JQ236861.1_Hu_COL1A1_REV
**25**	TGCTCTTTGTCTCGGACCAC	AB371583.1_Hu_COL5A1_FWD
**26**	TCCTTCCCTAGGTCTTCGGG	AB371583.1_Hu_COL5A1_REV
**27**	GAGAAGGCTGGGGCTCATTT	BC083511.1_Hu_GAPDH_FWD
**28**	AGTGATGGCATGGACTGTGG	BC083511.1_Hu_GAPDH_REV
**TaqMan Gene Expression Assays for RT-qPCR**		
**S. No.**	**Gene name**	**Assay ID (Thermo Scientific)**
**1**	*KERA*	Hs00559942_m1
**2**	*LUM*	HS00929860_m1
**3**	*PAX6*	Hs01088112_m1
**4**	*ACTA2*	Hs00426835_g1
**5**	*GAPDH*	Hs99999905_m1
**6**	*DCN*	Hs00754870_s1
**7**	*COL1A1*	Hs01076772_gH
**8**	*ALCAM*	Hs00977641_m1
**9**	*ATP1A1*	Hs00167556_m1
**10**	*CDH2*	Hs00983056_m1
**11**	*AQP1*	Hs01028916_m1
**12**	*TFAP2A*	Hs01029413_m1
